# Functional genomic analyses uncover *APOE*-mediated regulation of brain and cerebrospinal fluid beta-amyloid levels in Parkinson disease

**DOI:** 10.1186/s40478-020-01072-8

**Published:** 2020-11-19

**Authors:** Laura Ibanez, Jorge A. Bahena, Chengran Yang, Umber Dube, Fabiana H. G. Farias, John P. Budde, Kristy Bergmann, Carol Brenner-Webster, John C. Morris, Richard J. Perrin, Nigel J. Cairns, John O’Donnell, Ignacio Álvarez, Monica Diez-Fairen, Miquel Aguilar, Rebecca Miller, Albert A. Davis, Pau Pastor, Paul Kotzbauer, Meghan C. Campbell, Joel S. Perlmutter, Herve Rhinn, Oscar Harari, Carlos Cruchaga, Bruno A. Benitez

**Affiliations:** 1 Department of Psychiatry, BJC Institute of Health, Washington University School of Medicine, Box 8134, 425 S. Euclid Ave., St. Louis, MO 63110 USA; 2 NeuroGenomics and Informatics Center, Washington University, St. Louis, MO 63110 USA; 3 Hope Center for Neurologic Disorders, Washington University, St. Louis, MO 63110 USA; 4 Department of Neurology, Washington University, St. Louis, MO 63110 USA; 5 The Charles F. and Joanne Knight Alzheimer Disease Research Center, Washington University School of Medicine, St. Louis, MO 63110 USA; 6 Department of Pathology and Immunology, Washington University, St. Louis, MO 63110 USA; 7 College of Medicine and Health, University of Exeter, Exeter, Devon UK; 8 Memory Unit, Department of Neurology, University Hospital Mutua de Terrassa, University of Barcelona, Terrassa, Barcelona Spain; 9 Fundació per a la Recerca Biomèdica i Social Mútua de Terrassa, University of Barcelona, Terrassa, Barcelona Spain; 10 Departments of Neuroscience and Radiology, Programs in Physical Therapy and Occupational Therapy, Washington University, St. Louis, MO 63110 USA; 11 Department of Bioinformatics, Alector, INC, San Francisco, CA 94080 USA; 12 Department of Genetics, Washington University School of Medicine, St. Louis, MO 63110 USA

**Keywords:** Parkinson disease, GWAS, Alpha-synuclein, Amyloid beta, APOE

## Abstract

Alpha-synuclein is the main protein component of Lewy bodies, the pathological hallmark of Parkinson’s disease. However, genetic modifiers of cerebrospinal fluid (CSF) alpha-synuclein levels remain unknown. The use of CSF levels of amyloid beta_1–42_, total tau, and phosphorylated tau_181_ as quantitative traits in genetic studies have provided novel insights into Alzheimer’s disease pathophysiology. A systematic study of the genomic architecture of CSF biomarkers in Parkinson’s disease has not yet been conducted. Here, genome-wide association studies of CSF biomarker levels in a cohort of individuals with Parkinson’s disease and controls (N = 1960) were performed. PD cases exhibited significantly lower CSF biomarker levels compared to controls. A SNP, proxy for *APOE* ε4, was associated with CSF amyloid beta_1–42_ levels (effect = − 0.5, p = 9.2 × 10^−19^). No genome-wide loci associated with CSF alpha-synuclein, total tau, or phosphorylated tau_181_ levels were identified in PD cohorts. Polygenic risk score constructed using the latest Parkinson’s disease risk meta-analysis were associated with Parkinson’s disease status (p = 0.035) and the genomic architecture of CSF amyloid beta_1–42_ (R^2^ = 2.29%; p = 2.5 × 10^−11^). Individuals with higher polygenic risk scores for PD risk presented with lower CSF amyloid beta_1–42_ levels (p = 7.3 × 10^−04^). Two-sample Mendelian Randomization revealed that CSF amyloid beta_1–42_ plays a role in Parkinson’s disease (p = 1.4 × 10^−05^) and age at onset (p = 7.6 × 10^−06^), an effect mainly mediated by variants in the *APOE* locus. In a subset of PD samples, the *APOE* ε4 allele was associated with significantly lower levels of CSF amyloid beta_1–42_ (p = 3.8 × 10^−06^), higher mean cortical binding potentials (p = 5.8 × 10^−08^), and higher Braak amyloid beta score (p = 4.4 × 10^−04^). Together these results from high-throughput and hypothesis-free approaches converge on a genetic link between Parkinson’s disease, CSF amyloid beta_1–42_, and *APOE*.

**Electronic supplementary material:**

The online version of this article (10.1186/s40478-020-01072-8) contains supplementary material, which is available to authorized users.

## Introduction

Parkinson’s disease (PD) is a neurodegenerative disease characterized by rest tremor, rigidity, bradykinesia, and postural instability [57]. It is the most common neurodegenerative movement disorder, affecting more than six million people worldwide, with its prevalence projected to double in the next several decades [29]. Aggregated and phosphorylated alpha-synuclein (α-Syn) is the main protein component of Lewy bodies (LB) and neurites, the pathological hallmark of Lewy body diseases. The gene dosage effect of the *SNCA* gene, which encodes α-Syn, correlates with cerebrospinal fluid (CSF) α-Syn levels and a more severe PD phenotype [30, 55]. Common variants in the *SNCA* promoter are among the top genome-wide association studies (GWAS) signals for PD [54], suggesting that genetic control of CSF α-Syn level plays a role in PD phenotype variability. A modest but significant decrease (~ 10% to 15%) in CSF α-Syn levels has been reported in PD cases compared to controls [33] and is correlated with disease progression [1, 13, 35]. CSF α-Syn is not currently used as a clinical biomarker [35, 53], but is a proxy for pathological brain α-Syn accumulation [64]. Therefore, identifying genetic modifiers of CSF α-Syn levels could provide insight into PD pathogenesis. To date, genetic modifiers of CSF α-Syn remain unknown.

The α-Syn accumulation in specific brain regions defines different subtypes of Lewy body diseases (LBD). However, pure α-Syn pathology is only found in 45% (brainstem), 32% (limbic) and 19% (neocortical) of LBD. Concomitant presence of amyloid beta (Aβ), tau, and TDP-43 are common findings in LBD. Thus, Aβ and tau pathology is present in up to 80% and 53% in cases of neocortical LBD, respectively [58]. LBD patients with concomitant Alzheimer’s disease (AD) pathology exhibit a faster cognitive decline [44]. CSF levels of amyloid beta_1–42_ (Aβ42), total tau (t-tau), and phosphorylated tau_181_ (p-tau_181_) are commonly used as proxies of Aβ and tau pathology in the brain [8]. A correlation between lower Aβ42 CSF levels and higher Braak stage scores of AD neuropathology was found in neuropathologically confirmed LBD cases [8]. In cross-sectional studies, PD cases exhibit lower CSF levels of Aβ42 compared to age and gender-matched healthy controls [14, 52]. CSF levels of Aβ42 and t-tau levels are also associated with cognitive decline progression [52]. Decreased CSF Aβ42 levels predict the development of dementia in PD patients [47, 63]. These results suggest that dementia-associated CSF biomarker profile signatures could be informative of brain pathology in PD patients. GWAS using CSF Aβ42, t-tau, and p-tau_181_ levels as quantitative traits have identified genes involved in AD pathogenesis [24]. However, a systematic study of the role of genetic modifiers of dementia CSF biomarkers in PD has not yet been thoroughly evaluated.

This study aimed to uncover genetic modifiers of α-Syn, Aβ42, t-tau, and p-tau_181_ CSF levels in PD patients by performing a large (N = 1960) GWAS meta-analysis of CSF biomarkers in PD cohorts. Polygenic risk scores (PRS) and Mendelian randomization (MR) analyses were integrated with the latest PD risk meta-analysis (META-PD) and CSF biomarker summary statistics to examine the causal relationship between CSF biomarkers and PD risk. This is the first comprehensive analysis of CSF biomarkers using GWAS, PRS, and MR in PD.

## Materials and methods

### Study design

The goal of this study was to identify common genetic variants and genes associated with CSF α-Syn, Aβ42, tau, and p-tau_181_ in PD. A three-stage GWAS was used: discovery, replication, and meta-analysis. The discovery phase included 729 individuals from the Protein and Imaging Biomarkers in Parkinson’s disease study (PIB-PD) at the Washington University Movement Disorder Center [9] (n = 103) and the Knight ADRC [24] (n = 626). The replication phase included 1231 independent CSF samples obtained from PD cases and healthy elderly individuals from three additional studies [the Parkinson’s Progression Markers Initiative (PPMI), Alzheimer Disease Neuroimaging Initiative (ADNI), and Spain]. Meta-analyses were performed using a fixed-effects model. Genetic loci that passed the multiple test correction for GWAS (p < 5.0×10^−8^) were functionally annotated using bioinformatics tools to identify variants and genes driving the GWAS signal. PRS were used to test the correlation between CSF biomarkers and PD genetic architecture. Instrumental variables were selected from the summary statistics of CSF biomarkers, and MR methods were applied to test causality.

### Cohorts/datasets

This cross‐sectional multicenter study was performed using 1960 samples from non-Hispanic white (NHW) individuals from four cohorts: Washington University in Saint Louis (WUSTL) (N = 729), PPMI (N = 785), the University Hospital Mutua Terrassa (Spain, N = 130) and ADNI (N = 316). Cohorts included 700 clinically diagnosed PD cases, 564 controls, and 386 clinically-diagnosed AD cases. The remaining N = 310 individuals do not exhibit symptoms of neurodegenerative disease (Table 1 and Additional file 2: Table S1). PD clinical diagnoses were based on the UK Brain Bank criteria [39]. Clinical, biomarker, and genetic data from the PPMI and ADNI were obtained from the corresponding data repositories (www.ppmi-info.org and http://adni.loni.usc.edu/), accessed most recently on April 2019. The demographic characteristics of some of those cohorts have been published previously [4, 21, 28]. PPMI is a prospective study with ongoing recruitment. CSF samples were obtained at baseline (N = 510), 6 months (N = 385), and yearly after enrolment (N_1stYear_ = 428, N_2ndYear_ = 404, and N_3rdYear_ = 320). CSF α-Syn, Aβ42, t-tau, and p-tau_181_ were available for all the mentioned time points in the PPMI cohort.

**Table 1:** Summary demographics for the individuals with CSF measurements available

	All	PD cases	Controls	AD cases	Non-neurodegenerative^a^
N	1960	700	564	386	310
Age (years, 95% CI)	69.3 (62.0–75.0)	66.2 (59.6–73.3)	70.0 (64.9–74.3)	75.0 (70.0–80.0)	64.0 (55.0–73.0)
Males (N, %)	1107 (56.5%)	435 (62.1%)	297 (52.7%)	222 (57.5%)	153 (49.4%)
Alpha-Synuclein (ZScore(pg/mL))	− 0.02 (− 0.67 to 0.65)	− 0.03 (− 0.70 to 0.60)	0.02 (− 0.71 to 0.71)	0.14 (− 0.57 to 0.80)	− 0.23 (− 0.76 to 0.47)
Amyloid Beta (ZScore(pg/mL))	− 0.20 (− 0.72 to 0.64)	− 0.20 (− 0.72 to 0.45)	0.11 (− 0.52 to 1.04)	− 0.63 (− 1.00 to − 0.25)	0.03 (− 0.49 to 0.90)
Total Tau (ZScore(pg/mL))	− 0.27 (− 0.66 to 0.40)	− 0.27 (− 0.70 to 0.39)	− 0.34 (− 0.67 to 0.28)	0.22 (− 0.41 to 1.00)	− 0.50 (− 0.73 to − 0.08)
Phosphorylated Tau (ZScore(pg/mL))	− 0.25 (− 0.69 to 0.42)	− 0.30 (− 0.72 to 0.35)	− 0.27 (− 0.70 to 0.35)	0.22 (− 0.42 to 0.95)	− 0.48 (− 0.81 to − 0.04)

Concentration values have been standardized using ZScore for comparison purposes. Non-transformed values cannot be compared because there are several measuring methods: *SomaScan* platform, INNOTEST assay, xMAP-Luminex with INNOBIA AlzBio3, Roche Elecsys cobas e 601 and Euroimmun

^**a**^Includes individuals classified as not being Alzheimer’s disease, Parkinson’s disease or having dementia but neither controls such as essential tremor

### Biomarker measurements

α-Syn in CSF was measured in 107 samples from the WUSTL cohort [9] and the entire PPMI cohort, using a commercial ELISA kit (Covance, Dedham, MA) [45]. The additional samples (N = 622) from WUSTL were quantified using the *SOMAScan* platform (See below). Aβ42, t-tau, and p-tau_181_ were quantified using the INNOTEST assay (WUSTL) and xMAP-Luminex with INNOBIA AlzBio3 (PPMI). The immunoassay platform from Roche Elecsys cobas e 601 was used in the ADNI cohort to quantify all four biomarkers. ELISA assays from Euroimmun (Germany) were used in the Spanish cohort to measure the CSF levels of α-Syn, Aβ42, t-tau, and p-tau_181_. The α-Syn levels were normalized by log_10_ transformation. Aβ42, t-tau, and p-tau_181_ values were normalized and standardized by the Z score transformation. Individuals with biomarker levels outside three standard deviations of the mean were removed from the analysis (Table 1).

### Amyloid beta imaging

[11C]-Pittsburgh Compound B (PIB) acquisition and analysis were performed according to published methods [34]. Briefly, 10-15 mCi of the radiotracer was injected via an antecubital vein, and a 60-min, a three-dimensional dynamic PET scan was collected in 53 frames. Emission data were corrected for scattering, randoms, attenuation, and dead time. Image reconstruction produced images with a final resolution of 6 mm full-width half-maximum at the center of the field of view. Frame alignment was corrected for head motion and co-registered to each person’s T1-weighted magnetization-prepared rapid gradient echo magnetic resonance scan [61]. For quantitative analyses, three-dimensional regions of interest (prefrontal cortex, gyrus rectus, lateral temporal cortex, precuneus, occipital lobe, caudate nucleus, brainstem, and cerebellum) were created by a blinded observer for each subject based on the individual’s MRI scans, with boundaries defined as previously described [50]. Binding potentials (BP_ND_) were calculated using Logan graphical analysis, with the cerebellum as the reference tissue input function [49, 50]. Mean cortical binding potentials (MCBP) were calculated for each subject as the average of all cortical regions except the occipital lobe.

### Neuropathologic analysis

The neuropathological analysis was done at WUSTL, as previously reported [47]. Briefly, brains were fixed in 10% neutral buffered formalin for 2 weeks. Paraffin-embedded sections were cut at 6 μm. Blocks were taken from the frontal, temporal, parietal, and occipital lobes (thalamus, striatum, including the nucleus basalis of Meynert, amygdala, hippocampus, midbrain, pons, medulla oblongata) and the cervical spinal cord. Histologic stains included hematoxylin–eosin and a modified Bielschowsky silver impregnation. The Alzheimer’s disease pathologic changes were rated using an amyloid plaque stage (range, 0 to A–C) [7] and diffuse and neuritic plaques were also assessed. Cases were classified according to the neuropathologic criteria of Khachaturian [46], the Consortium to Establish a Registry for Alzheimer Disease (CERAD) [51] and NIA-Reagan [18].

### Genotyping

All cohorts, except PPMI, were genotyped using the Global Screening Array (GSA) Illumina platform. Genotyping quality control and imputation were performed using SHAPEIT [23] and IMPUTE2 [38] with the 1000 genomes as a reference panel. Single nucleotide polymorphisms (SNPs) with a call rate lower than 98% and autosomal SNPs that were not in Hardy–Weinberg equilibrium (p < 1.0×10^−06^) were excluded from downstream analyses. The X chromosome SNPs were used to determine sex based on heterozygosity rates. Samples in which the genetically inferred sex was discordant with the reported sex were removed. Whole-genome sequence data from the PPMI cohort was merged with imputed genotyped data; only variants present in both files were included in further analyses. Pairwise genome-wide estimates of proportion identity-by-descent tested the presence of unexpected duplicates and cryptically related samples (Pihat > 0.50). Unexpected duplicates were removed; the sample with a higher genotyping rate in the merged file was kept for those cryptically related samples. Finally, principal components were calculated using HapMap as an anchor. Only samples with European descent, an overall call rate higher than 95%, and variants with minor allele frequency (MAF) greater than 5% were included in the analyses.

### Single variant analysis

The three-stage single variant analysis was performed due to differences in time and platform for biomarker quantification. PLINK1.9 [16, 56] was used to perform the analysis of each cohort independently. A linear model using the normalized and standardized CSF levels and corrected by sex, age, and the first two principal components, was used. Disease status was not included in the model [25]. Then, the results for each protein were meta-analyzed using METAL [69]. For the α-Syn analyses, the WUSTL cohort was divided into two subsets based on the quantification method (ELISA or *SOMAscan)*.

### Analysis of variance

The genome-wide complex traits analysis (GCTA) software [71] was used to calculate the amount of variance explained by the *APOE* locus. GCTA estimates the phenotypic variance explained by genetic variants for a complex trait by fitting the effect of these SNPs as random effects in a linear mixed model.

### Multi-tissue analysis

The levels of α-Syn were measured in CSF, plasma, and brain (parietal cortex) using an aptamer-based approach (*SOMAScan* platform) [70]. After stringent quality control, CSF (n = 835), plasma (n = 529), and brain (n = 380) samples were included in the downstream analyses (Additional file 2: Table S2). The protein level was 10-based log-transformed to approximate the normal distribution and used as phenotype for the subsequent GWAS. The single variant analysis was performed in each tissue independently using PLINK1.9 [56]. A multi-tissue analysis using the multi-trait analysis of GWAS (MTAG) [66] was applied to increase the power of detecting a no tissue-specific protein quantitative trait loci for α-Syn. MTAG calculates the trait-specific effect estimate for each tissue separately and then performs a meta-analysis while accounting for sample overlap. Measurements of Aβ42, t-tau, and p-tau_181_ were not available in different tissues.

### Polygenic risk score

PRS is constructed by summing all trait-associated alleles in a target sample (META-PD and CSF biomarkers separately), weighted by the effect size of each allele in a base using different p-value thresholds. SNPs in linkage disequilibrium (LD) are grouped together to avoid extra weight into a single marker. The optimal threshold is considered the one that explains the maximum variance in the target sample. The association was tested using the default parameters and nine p-value cutoffs. The PRSice2 software [17] was used to calculate the PRS. Longitudinal measures of CSF α-Syn, Aβ42, t-tau, and p-tau_181_ were available for the PPMI cohort. A simplified PRS (detailed below) was used to test if the genetic architecture of PD was predictive of biomarker level progression. The PD PRS using sentinel SNPs from the META-PD [54] was modeled using the method previously described [19, 41, 42, 68]. Briefly, only genetic variants corresponding to the top hit on each GWAS locus (also known as sentinel SNP) available in the dataset with a minimum call rate of 85% were included in the PRS. If not possible, a proxy with R^2^ > 0.90 was used. The weight of each variant was calculated using the binary logarithm transformation of the reported Odd ratios. The final PRS is the sum of the weighted values for the alternate allele of all the sentinel SNPs.

### Mendelian randomization

MR requires that the genetic instruments are associated with the modifiable exposure of interest (GWAS of CSF biomarkers), and any association between the instruments and the outcome (PD risk) is mediated by the exposure [11]. A two-sample MR was used to estimate causal effects using the Wald ratio for single variants along with an inverse-variance–weighted (IVW) fixed-effects meta-analysis for an overall estimate [36]. The IVW estimate is the inverse variance weighted mean of ratio estimates from 2 or more instruments. Two-sample MR provides an estimate of the causal effect of an exposure on an outcome, using independent samples to obtain the gene-exposure and gene-outcome associations, provided three key assumptions: (i) genetic variants are robustly associated with the exposure of interest (i.e. replicate in independent samples), (ii) genetic variants are not associated with potential confounders of the association between the exposure and the outcome and (iii) there are no effects of the genetic variants on the outcome, independent of the exposure (i.e. no horizontal pleiotropy). To account for potential violations of the assumptions underlying the IVW analysis, a sensitivity analysis using MR-Egger regression and the weighted median estimator was performed [36]. MR Egger regression consists of a weighted linear regression of SNP META-PD against SNP biomarker effect estimates. Assuming that horizontal pleiotropic effects and SNP exposure associations are uncorrelated (i.e., the instrument strength independent of direct effects assumption), MR Egger regression provides a valid effect estimate even if all SNPs are invalid instruments. Moreover, the MR Egger intercept can be interpreted as a test of overall unbalanced horizontal pleiotropy because one would expect a null y-intercept (i.e., the mean value of the SNP META-PD associations when the SNP biomarker association is zero) if there are no horizontal pleiotropic effects. Robust regression to downplay the contribution to the causal estimate of instrumental variables with heterogeneous ratio estimates were also performed [10, 12]. Heterogeneity (i.e., instrument strength) was tested using the I^2^ statistic. I^2^ statistic, instead of F statistic, is a better indicator of instrument strength for the two-sample summary data approach [6]. The R package “MendelianRandomization” [72] (version 0.4.1) was used for the MR analyses.

The latest and largest meta-analysis for PD genetic risk was used to perform the MR analyses [54]. Summary statistics from the largest GWAS of CSF Aβ42, t-tau, and p-tau_181_ were also used [25]. Deming et al. performed a one-stage GWAS for 3146 NHW individuals across nine independent studies [25]. None of these cohorts included PD affected individuals for each biomarker (Aβ42, t-tau, and p-tau_181_). Finally, the summary statistics of the GWAS for α-Syn CSF levels generated in the current study were used. There was no overlap between CSF biomarker datasets and PD risk datasets. Instrumental variables for each GWAS were obtained by clumping each GWAS summary statistics based on the LD structure of the exposure (CSF biomarker levels) and a significance threshold of 1.0x10^−5^ using PLINK1.9 [56]. Instrumental variables were restricted to those that are uncorrelated (in linkage equilibrium) by setting the –clump-r2 flag to 0.0 and the –clump-kb flag to 1000 (1 Mb).

## Results

### Association of CSF biomarkers with disease status

**Fig. 1:**
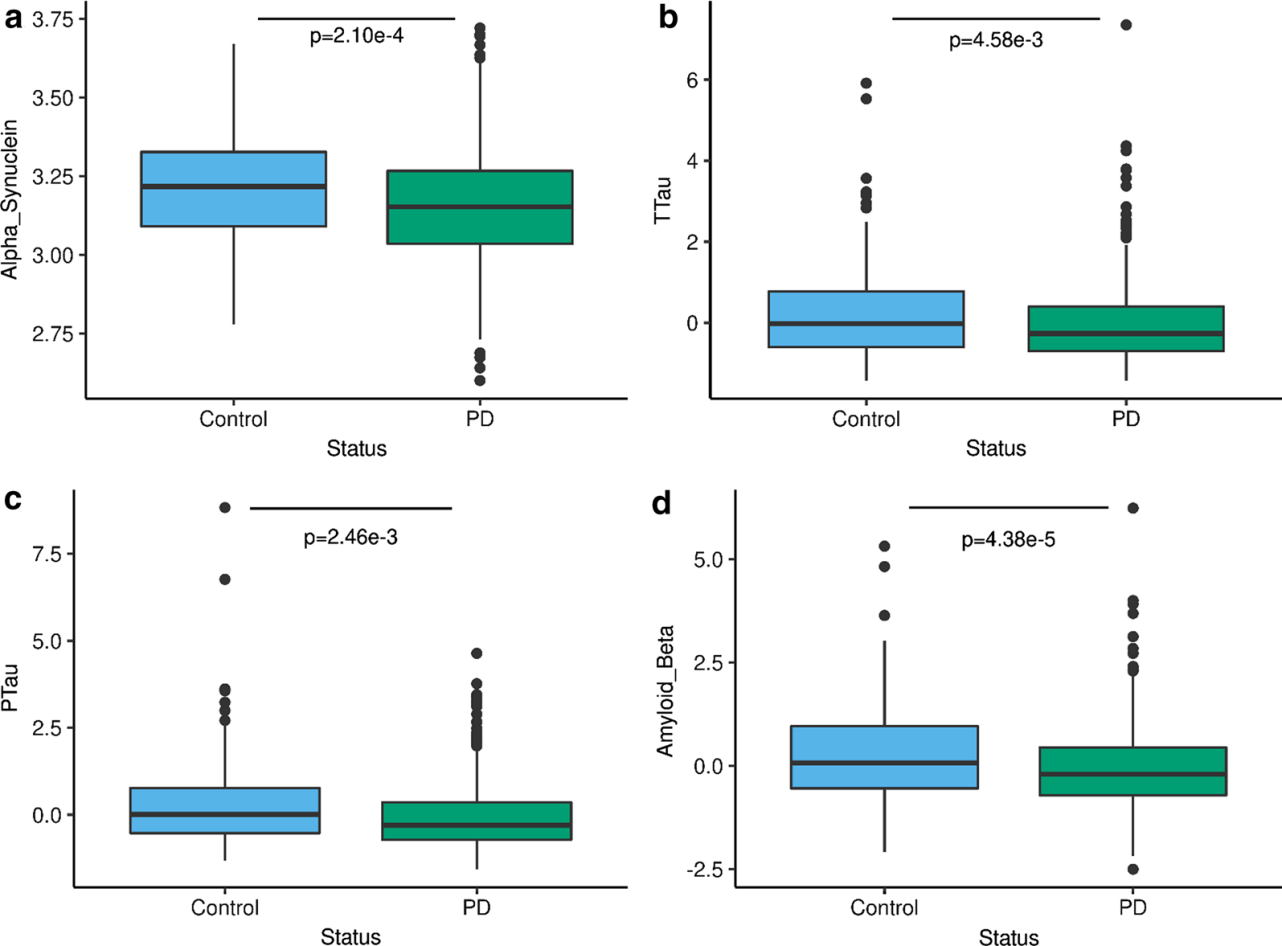
CSF α-Syn, Aβ42, t-tau, and p-tau_181_ levels are lower in Parkinson’s disease than in controls. Box plot of the normalized CSF levels of **a** α-Syn. **b** total tau. **c** phosphorylated tau and **d** Aβ42 in controls (gray) and Parkinson’s disease cases (orange). Parkinson’s disease cases (N = 700) and controls (N = 189) from two independent datasets (WUSTL and PPMI). The means for each group are represented by a horizontal line. A generalized linear model (CSF biomarker levels ~ Age + Sex + Status) was used to calculate the statistical differences between the CSF protein levels in Parkinson’s disease cases and controls

A generalized linear model (CSF biomarker levels ~ Age + Sex + Status) including PD cases (N = 700) and controls (N = 189) from two independent datasets (WUSTL and PPMI—Additional file 2: Table S1) in which α-Syn levels were measured with the same platform revealed that all CSF biomarker levels were significantly lower in PD cases compared to controls (α-Syn: beta_PD_ = − 0.05, p = 2.10 × 10^−04^; Aβ42: beta_PD_ = − 0.34, p = 4.38 × 10^−05^; t-tau: beta_PD_ = − 0.23, p = 4.58 × 10^−03^; and p-tau_181_: beta_PD_ = − 0.25, p = 2.46 × 10^−03^—Fig. 1). All associations passed multiple test correction (p < 0.013). Using a longitudinal model adjusted by age at lumbar puncture, sex, and the first two principal components, we found significant changes over time for CSF Aβ42 (p = 0.01) but not for α-Syn, t-tau, or p-tau_181_ in the PPMI cohort (N = 785). These results suggest that CSF dementia biomarkers are associated with PD status.

### No significant loci were identified for CSF α-Syn, t-tau or p-tau_181_ in Parkinson’s disease cohorts

**Fig. 2:**
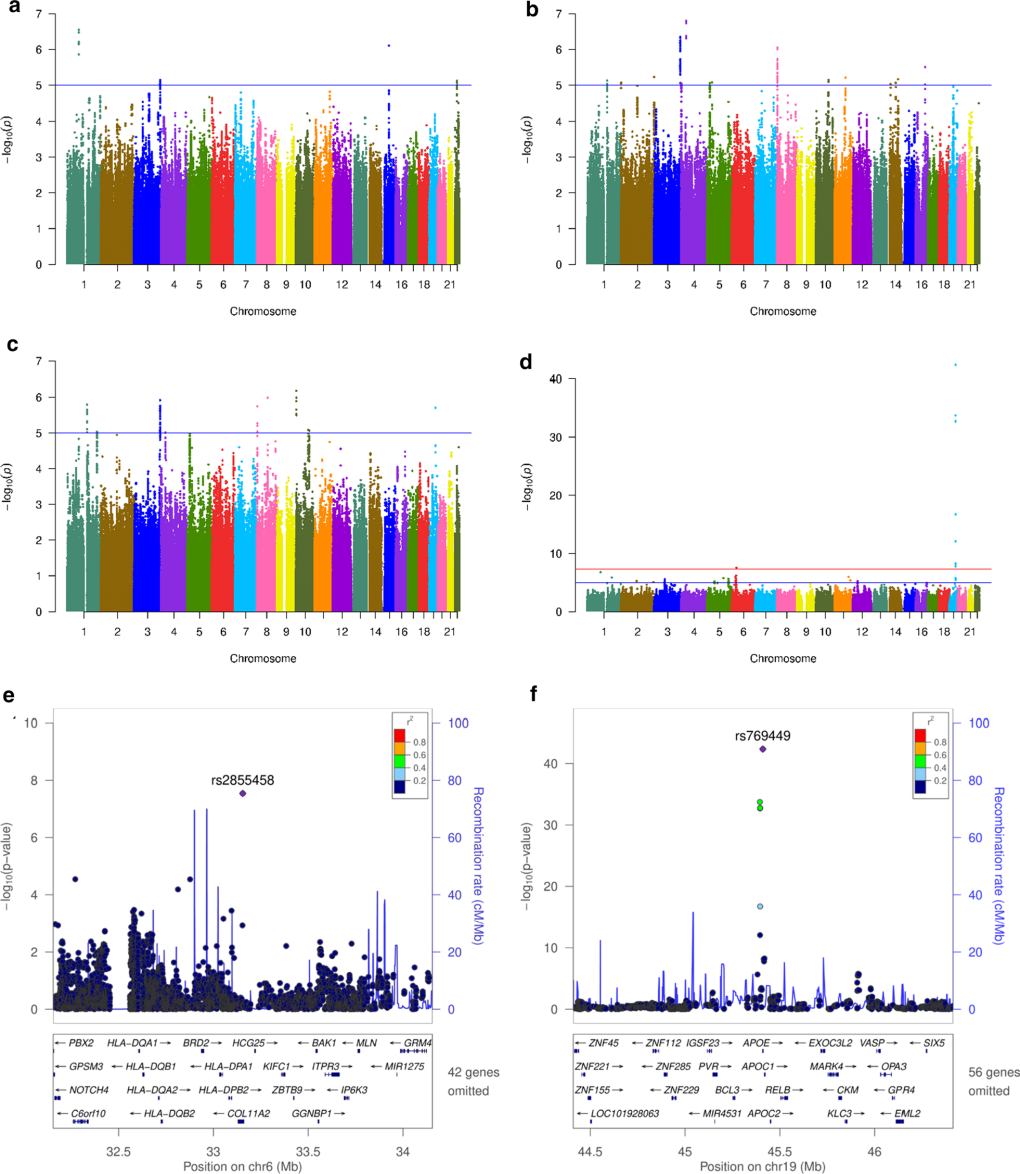
Association plot of single variant analyses of CSF α-Syn, t-tau, p-tau_181_ and Aβ42 levels. Manhattan plot shows negative log_10_-transformed p-values from the meta-analysis of **a** α-Syn. **b** total tau. **c** phosphorylated tau and **d** Aβ42 CSF levels. The lowest p-value on chr19 (*APOE* locus) was p = 4.5 × 10^−43^. The horizontal lines represent the genome-wide significance threshold, p = 5×10^−8^ (red) and suggestive threshold, p = 1×10^−5^ (blue). **e**, **f** Regional association plots of loci are shown for SNPs associated with CSF Aβ42 levels near *HLA* (**e**) and near *APOE* locus (**f**). The SNPs labeled on each regional plot had the lowest p-value at each locus and are represented by a purple diamond. Each dot represents an SNP, and dot colors indicate linkage disequilibrium with the labeled SNP. Blue vertical lines show the recombination rate marked on the right-hand y-axis of each regional plot. Suggestive SNPs for α-Syn, t-tau, p-tau_181_ can be found in Additional file 2: Tables S3 to S6

Within each cohort, a linear regression testing the additive genetic model of each SNP for association with CSF protein levels using age, gender, and two principal component factors for population stratification as covariates did not reveal any genome-wide significant loci associated with CSF α-Syn. Although several suggestive loci (p < 10^−6^ to 10^−8^) were identified in these analyses (Additional file 1: Fig. S1 and Additional file 2: Table S3), none of them passed multiple test correction threshold when cohorts were combined in the meta-analysis (Fig. 2a and Additional file 2: Table S3).

Joint analysis for CSF α-Syn levels stratifying by PD cases (N = 700), PD cases and controls (N = 889), AD cases only (N = 386), AD cases and controls (N = 575) and controls only (N = 189) were also performed. None of these analyses revealed any genome-wide significant locus, suggesting that these sample sizes might be underpowered to uncover the genetic modifiers of CSF α-Syn.

For t-tau, individual cohort analyses revealed four genome-wide significant loci (Additional file 1: Fig. S3 and Additional file 2: Table S5). However, none of them remained significant in the meta-analyses (Fig. 2b and Additional file 2: Table S5). For p-tau_181_, individual cohort analyses revealed three genome-wide significant loci (Additional file 1: Fig. S4 and Additional file 2: Table S6). However, none achieved significance in the meta-analyses (Fig. 1c and Additional file 2: Table S6).

### Genetic analyses of multi-tissue α-Syn levels

In a subgroup of samples, α-Syn levels were measured in plasma (N = 529), brain (N = 380), and CSF (N = 835) using the *SOMAScan* platform (Additional file 2: Table S2). Single variant analysis was performed in each tissue separately (Additional file 1: Fig. S2A to 2C). Multi-tissue analysis was performed using MTAG [66]. Although two suggestive loci were observed in chromosomes 3 and 13 (Additional file 1: Fig. S2D and Additional file 2: Table S4) within genomic regions enriched with long intergenic non-protein coding (LINC) genes (Additional file 1: Fig. S2E and F), no genome-wide significant locus was identified. These results suggest that the power boost of using MTAG is not enough to unveil the genetic architecture of α-Syn.

### *APOE* locus is associated with Aβ42 CSF levels in Parkinson’s disease cohorts

A proxy SNP for *APOE* ε4, rs769449, was associated with CSF levels of Aβ42 in the WUSTL (effect = − 0.56, p = 4.15 × 10^−19^), and ADNI cohorts (effect = − 0.73, p = 1.25 × 10^−15^). This association did not pass the genome-wide multiple test correction threshold in the PPMI cohort (effect = − 0.43, p = 3.09 × 10^−07^) and was not significant in the Spanish cohort (Additional file 1: Fig. S5 and Additional file 2: Table S7). The *APOE* locus (effect = − 0.57, p = 4.46 × 10^−43^) and a locus in the *HLA* region (effect = 0.23, p = 2.88 × 10^−08^) remained significant in the meta-analysis (Fig. 2d–f). When the cohorts containing only PD cases and controls were analyzed jointly (WUSTL and PPMI – N = 700 cases and 189 controls), the *APOE* locus was GWAS significant (effect = − 0.50, p = 9.25 × 10^−19^) but not the *HLA* region (effect = 0.22, p = 3.58 × 10^−04^). In the combined analysis of all cohorts (N = 1960), the *APOE* locus accounted for 36.2% of the CSF Aβ42 levels variance (p = 2.35 × 10^−03^). Overall, these results revealed a strong and highly significant association between *APOE* locus and lower CSF Aβ42 levels in PD cohorts.

### Significant correlation of genomic architecture of Parkinson’s disease risk and CSF Aβ42

**Fig. 3:**
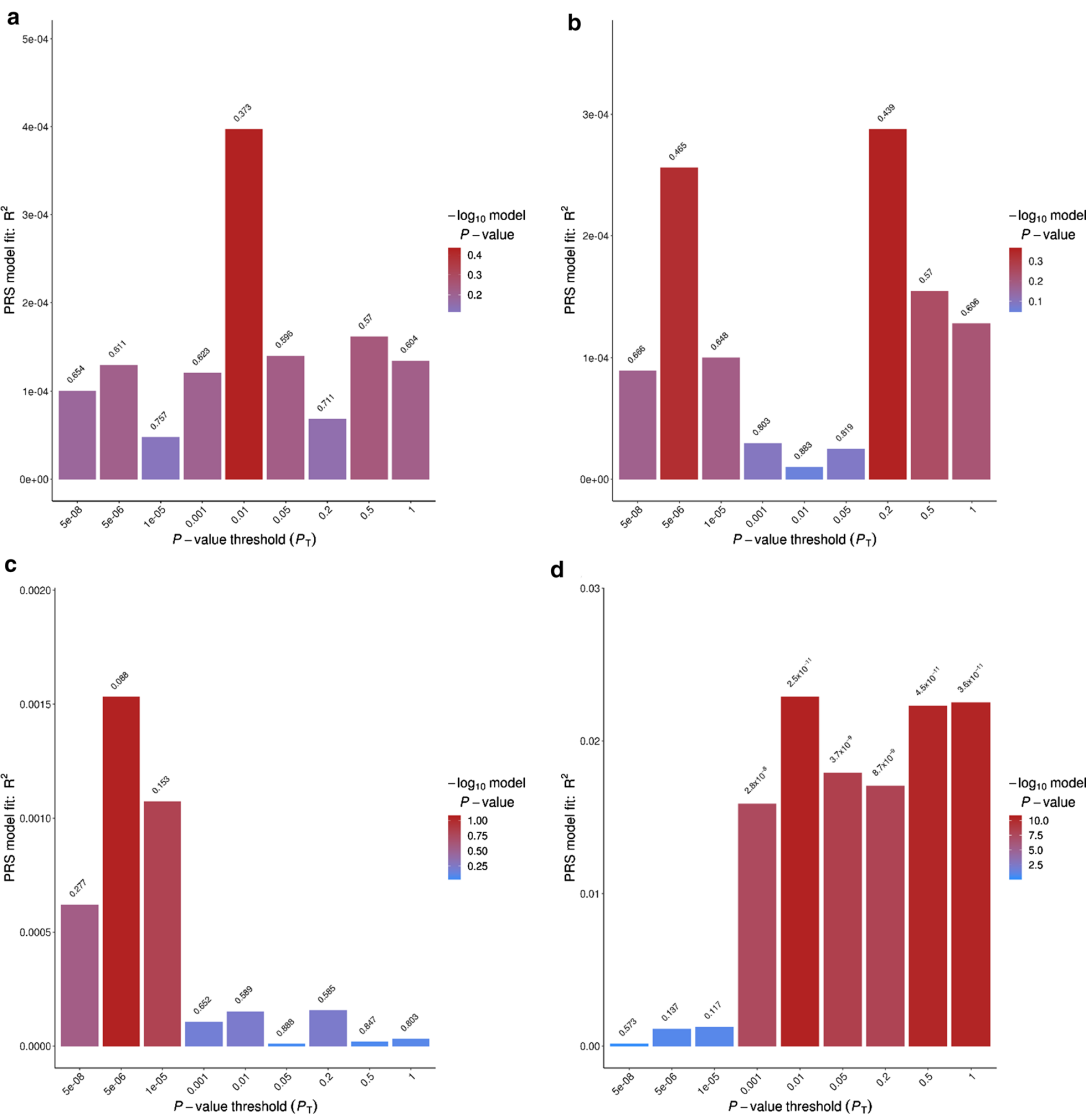
Genetic architecture correlations of Parkinson’s disease risk with CSF α-Syn, t-tau, p-tau_181_ and Aβ42 levels. PRSice bar plots for Parkinson’s disease risk and CSF biomarkers. Nagelkerke pseudo-R-squared fit for the model of **a** CSF α-Syn levels PRS and Parkinson’s disease risk. **b** CSF t-tau levels PRS and Parkinson’s disease risk. **c** CSF p-tau_181_ PRS and Parkinson’s disease risk. **d** CSF Aβ42 levels PRS and Parkinson’s disease risk. Total variance explained by the PRS for multiple p-value thresholds for the inclusion of SNPs, with the red bar indicating the optimal p-value threshold (P_T_), explaining the maximum amount of variance (R^2^) in Parkinson’s disease risk in the target sample

PRS at different p-value thresholds were used to test if the genetic variants associated with dementia biomarkers were associated with the genomic architecture of PD. PRS calculated using the META-PD [54] were associated with PD status in the WUSTL cohort (N = 108; p = 0.035). The PPMI cohort was excluded from this analysis due to overlap with META-PD. No correlation was observed between the genetic architecture of PD and that of CSF α-Syn, t-tau, or p-tau_181_ levels (Fig. 3). In contrast, the genetic architecture of CSF Aβ42 was correlated with PD, with the best fit when collapsing independent SNPs with p-value < 0.01 (p = 2.50 × 10^−11^) with a correlation coefficient (R^2^) of 2.29%. In PD cases and controls only, the correlation remained significant (p = 4.78 × 10^−08^), with an R^2^ of 2.36%. In PD patients with both GWAS and CSF biomarker data, the CSF levels of each biomarker were analyzed by quartiles of the PRS calculated from META-PD risk. A significant difference (p = 7.30 × 10^−04^) was found among the top and the bottom quartiles; higher PRS values exhibit lower levels of CSF Aβ42 (Additional file 1: Fig. S6). No association between PD PRS and longitudinal changes of α-Syn, Aβ42, t-tau, and p-tau_181_ levels was found in the PPMI dataset. These results indicate that PD and Aβ42 CSF levels have a shared genomic architecture.

### Mendelian randomization suggest a causal link between CSF Aβ42 and Parkinson’s disease

**Fig. 4:**
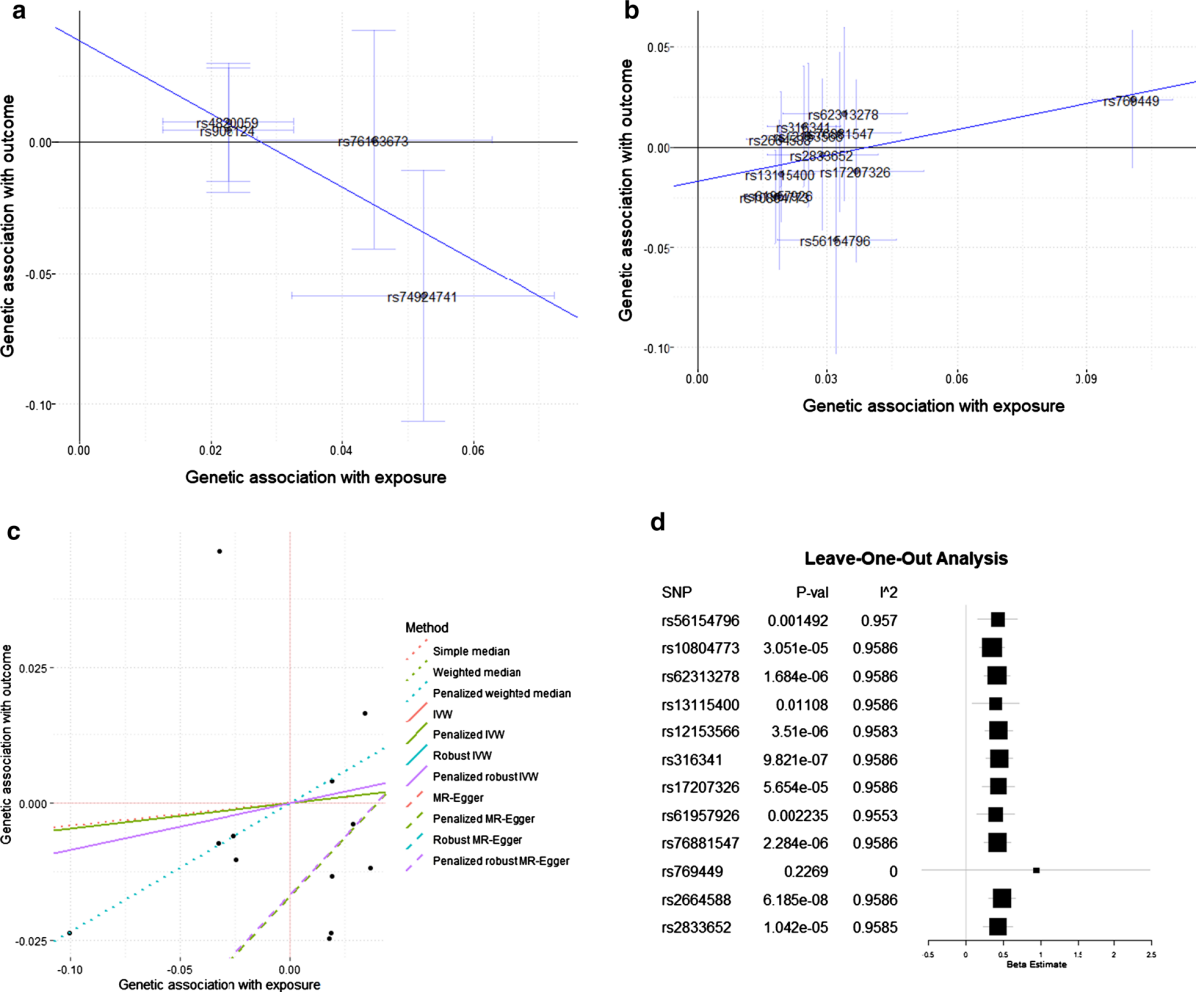
MR regressions on Parkinson’s disease risk genetic architecture and CSF α-Syn and Aβ42 levels. **a** Association between META-PD risk and CSF α-Syn levels (four variants). Robust regression MR-Egger method effect = -1.40 and p = 0.06, which is not consistent with causality. **b** Association between Parkinson’s disase risk and CSF Aβ42 levels (twelve variants). Robust regression with MR-Egger method effect = 0.43 and p = 1.44 × 10^−05^, which is consistent with causality. Each dot corresponds to one genetic variant, with a 95% confidence interval (CI) of its genetic association with the exposure (α-Syn and Aβ42 levels) and the outcome (Parkinson’s disease risk). Regression lines correspond to the robust MR-Egger method regression; numerical results are given for all tested methods in Additional file 2: Table S8. **c** CSF Aβ42 regression using multiple MR methods. Each dot is one of the twelve variants included in this test; the effect of CSF Aβ42 levels on the x-axis and Parkinson’s disease risk on the y-axis. Each line represents the regression of one MR-method of CSF Aβ42 levels on Parkinson’s disease risk with one MR method. Additional details on the data sources and analysis methods to generate these figures are provided in Additional file 2: Table S8. **d** The forest plot illustrates the leave-one-out sensitivity analysis between CSF Aβ42 and META-PD risk. MR analysis without rs769449 decreased the I^2^ statistic (I^2^ = 0.0%) and increased the p-value to non-significant levels, suggesting that the association is mainly driven by this variant

Robust regression with the MR-Egger method found no association for t-tau or p-tau_181_ levels but revealed a trend for CSF α-Syn levels (effect = − 1.40; p = 0.06), and a significant causal effect for CSF Aβ42 on PD (effect = 0.43; p = 1.44 × 10^−05^) (Fig. 4a–c and Additional file 2: Table S7; Table 2 and Additional file 2: Table S8). When each cohort included in the META-PD was tested separately, CSF Aβ42 showed a causal effect in Nalls et al., 2014 and 2019 (p = 1.54 × 10^−07^ and 8.74 × 10^−05^ , respectively), but not in Chang et al., 2017 (Table 2 and Additional file 2: Table S8). Additionally, a significant causal effect for CSF Aβ42 on PD age-at-onset was found using the data from Blauwendraat et al., 2019 (effect = 7.75; p = 7.65 × 10^−06^—Table 2 and Additional file 2: Table S8). A leave-one-out sensitivity analysis on CSF Aβ42 revealed that the proxy SNP for *APOE* ε4, rs769449 is the strongest instrumental variable of this analysis (I^2^ is greater than 90% except when this variant was removed) and the main driver of the causal effect of CSF Aβ42 on PD. Other SNPs contribute in a smaller proportion to the causal effect (Fig. 4d). Altogether these results suggest a causal role of SNPs on the *APOE* locus and CSF Aβ42 on PD.

**Table 2:** Mendelian randomization results for the causal role of α-Syn, Aβ42, tau, and t-tau in Parkinson’s disease using the robust regression MR-Egger method with robust regression

Biomarker	PD Risk1		PD age at Onset2	
	Effect	P-value	Effect	P-value
Alpha-synuclein	− 1.389	0.064	− 11.018	0.835
Amyloid-beta	0.430	1.44 × 10^−05^	7.746	7.65 × 10^−06^
Total tau	− 0.338	0.246	11.276	0.069
Phosphorylated tau	− 0.096	0.785	− 0.298	0.912

### *APOE* ε4 is associated with Aβ deposition in brains of Parkinson’s disease individuals

**Fig. 5:**
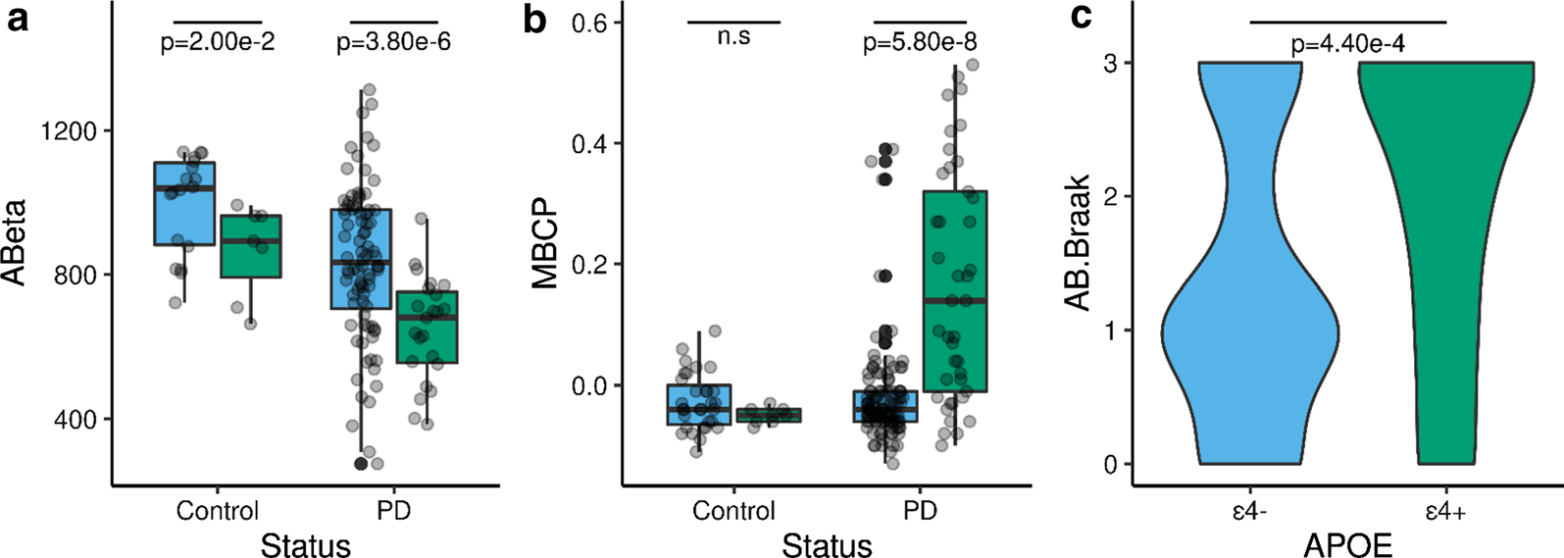
*APOE* ε4 is associated with Aβ42 deposition in the brains of Parkinson’s disease individuals. **a** Comparison of the levels of CSF Aβ42 in control (N = 26) and PD (N = 108) participants stratified by the presence (ε4 + ; green) or absence (ε4-; blue) of the *APOE* ε4 allele. **b** Effect of *APOE* ε4 allele on the levels of mean cortical binding potentials (MCBP) in controls (N = 44) and Parkinson’s disease (N = 156). **c** PD patients carrying the *APOE* ε4 allele exhibit a higher Braak Aβ score than non-carriers (N = 92). Differences between *APOE* ε4 carriers and non-carriers were statistically significant by the Mann–Whitney U test

CSF Aβ42 and *APOE* genotype data were available for 134 participants (N_Controls_ = 26 and N_Cases_ = 108). No difference in the *APOE* ε4 frequency was found between cases (0.14%) and controls (0.11%). However, the CSF Aβ42 levels were significantly different between controls (p = 3.00 × 10^−02^) and cases (p = 3.80 × 10^−06^) when stratifying by the presence of *APOE* ε4 allele (Fig. 5a) [9].

PET PiB analysis (N = 108) revealed that MCBP increased with age-at-onset (r = 0.20, p = 3.00 × 10^−02^) and number of *APOE* ε4 alleles (r = 0.22, p = 8.00 × 10^−03^) (Fig. 5a), but decreased CSF Aβ42 (r = − 0.55, p = 3.33 × 10^−12^). A linear regression model indicated that CSF Aβ42 and *APOE* ε4, explain 48% of the variance of MCBP. *APOE* ε4 is also significantly associated with MCBP (β = 0.14, p = 1.40 × 10^−06^) in analysis with 200 participants that included sex and age as covariates. *APOE* ε4 and age at onset explain 20% of the MCBP variance in this larger cohort. The presence of *APOE* ε4 did not affect the MCBP in controls (p = 0.19). However, PD patients carrying *APOE* ε4 exhibit significantly (p = 5.80 × 10^−08^) higher levels of MCBP than non-carriers (Fig. 5b).

Neuropathological data and *APOE* genotype were available from 92 PD cases. Individuals carrying an *APOE* ε4 allele had significantly (p = 4.40 × 10^−04^) higher Braak Aβ stage (Fig. 5c). *APOE* ε4 correlated with Braak Aβ stage (r = 0.33, p = 1.00 × 10^−03^) and diffuse plaques (r = 0.42, p = 5.00 × 10^−03^), but not with neuritic plaques (r = 0.42, p = 0.12). The best multiple linear regression model for the Braak Aβ stage, which included age at onset and *APOE* ε4, explained 42% of the variance of the Braak Aβ stage. Altogether, these results suggest that *APOE* ε4 drives the Aβ deposition in PD participants.

## Discussion

CSF α-Syn, Aβ42, t-tau, and p-tau_181_ levels were significantly lower in PD cases compared with controls, as we previously reported with a smaller sample size [9]. GWAS were performed using CSF biomarker levels as quantitative traits in a large cohort (N = 1,960). With the current sample size, no signal was below the GWAS significant threshold for CSF α-Syn, t-tau, or p-tau_181_. A SNP proxy for *APOE* ε4 was genome-wide associated with CSF Aβ42 levels. The PRS calculated using META-PD was associated with PD status and correlated with the genomic architecture of CSF Aβ42; in fact, individuals with higher PRS scores exhibit lower CSF Aβ42 levels. Two-sample MR analysis revealed that CSF Aβ42 probably plays a role in PD and PD age-at-onset, an effect mainly mediated by variants in the *APOE* locus. Using a subset of participants from the WUSTL cohort with additional clinical and neuropathological data, we found that the *APOE* ε4 allele was associated with lower levels of CSF Aβ42, higher cortical binding of PiB PET and higher Braak Aβ score.

This is the first comprehensive analysis of CSF α-Syn and AD biomarkers using GWAS, PRS, and MR in PD. We found lower levels of CSF α-Syn in PD cases compared to controls in a cross-sectional analysis but no significant differences in the longitudinal study (PPMI). CSF α-Syn, as measured with ELISA-based assays, is not a clinically useful diagnostic marker for PD, and utility as an outcome measure for clinical trials or progression is still controversial [35, 53]. CSF biomarkers in AD used as quantitative endophenotypes have provided insights into AD pathophysiology [24]. Here, we used a large CSF α-Syn cohort (N = 1920) to identify its genetic modifiers. However, we did not find any locus associated with CSF α-Syn levels. Recently, a GWAS on CSF α-Syn using the ADNI cohort (N = 209) reported a genome-wide significant locus [73] (rs7072338). In the present meta-analyses (N = 1960), the p-value for rs7072338 was not significant (0.99). In the ADNI cohort, we found a nominal association for this SNP (p = 0.50 × 10^−3^). No correlation was found between the genetic architecture of PD with cross-sectional or longitudinal CSF α-Syn levels, consistent with what we have previously reported [41]. Using MR methods, we found a trend for the association between the CSF α-Syn levels and the risk of developing PD. However, sensitivity analyses showed limited power due to the small number of variants included in the analyses.

MR analyses suggest that Aβ42 could play a causal role in PD. Our MR results consistently identified a causal correlation between the *APOE* locus, CSF Aβ levels, and PD. MR is used to test if the genetic variation associated with a trait has a causal relationship with a health outcome [20]. MR is not affected by confounding factors or reverse causation, like in observational studies. However, the proper implementation of MR depends on several assumptions [20]. Here, instrumental variables (SNPs) relevant to CSF Aβ were previously and consistently identified [24]. A second MR assumption is independence; SNPs associated with the trait (e.g. *APOE* locus with CSF Aβ) should not be associated with the outcome (PD risk). The third MR assumption is the exclusion restriction, which means that SNPs do not affect PD risk except through CSF Aβ levels. The two sample MR used here requires two additional criteria: both cohorts must have similar genetic background but no overlap with each other. Here, samples used for the MR analysis, summary statistics from Deming, et al. [24] and Nalls, et al. [54], met both criteria. We could not rule out a horizontal pleiotropic effect of all the SNPs associated with CSF Aβ with PD, but our study is powered to detect the causal association with the *APOE* locus. Thus, we inferred that the lifetime effect of the *APOE* locus is causal in relation to PD.

The *APOE* locus and CSF Aβ42 levels were GWAS significant in the meta-analysis. The association of the *APOE* locus with CSF Aβ42 levels has been previously reported in AD [48] but not in PD cohorts. Interestingly, the direction of the effect was the same as what has been reported in AD but with a higher effect size (− 0.57 in PD compared to − 0.10 in AD) [25]. The *APOE* locus is the most significant locus associated with sporadic LBD, [59, 60], and cognitive decline in PD [40], but not with PD risk [54]. Here, we also found for the first time that patients with higher PRS from PD risk exhibit lower levels of CSF Aβ42, suggesting that similar genes or pathways predispose individuals to an accumulation of Aβ in the brain and to develop PD. This is in agreement with a recent report suggesting that the PD genes from the PRS analysis are enriched for AD genes [2].

The results from unbiased analyses like GWAS, PRS and MR demonstrated a link between PD genetic risk with CSF Aβ42 levels and the *APOE* locus. Here, we also provided further evidence by showing that PD patients carrying the *APOE* ε4 allele presented with lower levels of CSF Aβ42 (p = 3.8 × 10^−06^), higher MCBP (p = 5.80 × 10^−08^) and higher Braak Aβ scores (p = 4.40 × 10^−04^). These results support the synergistic relationship between α-Syn and Aβ pathology in AD, PD and LBD brains [43], and the effect of Aβ plaques exacerbating the propagation of α-Syn pathology in mouse models [3]. It is known that *APOE* ε4 drives the production of Aβ, the accumulation of Aβ fibrils in AD patients [37], exacerbates tau-mediated neurodegeneration in a mouse model of tauopathy [62] and affects CSF αSyn levels in the prodromal phase of sporadic and familial AD [67]. However, the role of *APOE* in human synucleinopathies is probably more complex. In LBD patients, the *APOE* ε4 effect on α-Syn pathology could be dependent on concurrent Aβ and/or tau pathology [58], however *APOE* ε4 also promotes α-Syn pathology independently [27, 65] and affects CSF αSyn levels [67]. We recently showed that *APOE* ε4 increased the α-Syn phosphorylation, worsened motor impairment, and increased neuroinflammation and neurodegeneration in different mouse models [22].

This is the largest sample size used for discovering CSF α-Syn genetic modifiers to date and yet no GWAS significant locus was found. It is possible that the complexity of α-Syn genetic architecture makes the current sample size insufficiently powered to detect signals with a smaller effect. Here, we found lower levels of CSF α-Syn in PD patients, which aligns with previous reports. However, neither PRS nor MR analysis revealed evidence of the causal link of CSF α-Syn with PD risk. In fact, it has been reported that α-Syn aggregation is neither necessary nor sufficient for neurodegeneration or clinical parkinsonism [31, 32]. The cohorts used in this study rely on clinical diagnosis rather than neuropathological confirmation, which precludes analyses of a correlation between CSF α-Syn levels and pathologic brain accumulation of brain α-Syn. Factors that may have contributed to the lack of power to detect genetic modifiers of CSF α-Syn include participant characteristics (PD subtypes, misdiagnosis, comorbidities, medications, disease duration), preanalytical factors (blood contamination at lumbar puncture), and differences in assays (measuring various abnormal pathological or normal forms of α-Syn) [26].

PD is a heterogeneous disorder with different identifiable clinical-pathological subtypes based on symptom severity and predominance [15]. It is conceivable that more homogeneous PD subtypes could be defined using biomarker-driven, clinical-molecular phenotyping approaches. This study, with 1960 samples with CSF α-Syn levels, showed that the genomic architecture of α-Syn is complex and not correlated with the genomic landscape of PD. Additional studies with larger sample sizes and standardized methods to quantify α-Syn in both CSF and brain are needed to uncover genetic modifiers of α-Syn levels. Our results using high-throughput and hypothesis-free, unbiased approaches demonstrated a link between PD genetic risk, CSF Aβ42 levels and *APOE* locus. These findings were further validated by strong significant associations of *APOE* ε4 with Aβ deposition in cortical regions of living and postmortem PD patients.

## Supplementary information

**Additional file 1.** Supplemental Figures.

**Additional file 2.** Supplemental Tables.

## Data Availability

All data is available in the Center for Neurogenomics and informatics (NGI) website (https://neurogenomics.wustl.edu/). The summary statistics for all the analyses can be easily explored in the Online Neurodegenerative Trait Integrative Multi-Omics Explorer (ONTIME) (https://omics.wustl.edu) and the Charles F. and Joanne Knight Alzheimer Disease Research Center (https://knightadrc.wustl.edu/research/resourcerequest.htm).

## References

[1] Atik A, Stewart T, Zhang J (2016). Alpha-synuclein as a biomarker for Parkinson’s disease. Brain Pathol.

[2] Bandres-Ciga S, Saez-Atienzar S, Kim J, Makarious M, Faghri F, Diez-Fairen M, Iwaki H, Leonard H, Botia J, Ryten M, Hernandez D, Gibbs J, Ding J, Gan-Or Z, Noyce A, Pihlstrom L, Torkamani A, Scholz S, Traynor B, Ehrlich D, Scherzer C, Bookman M, Cookson M, Blauwendraat C, Nalls M, Singleton A (2020). Large-scale pathway-specific polygenic risk, transcriptomic community networks and functional inferences in Parkinson disease. bioRxiv.

[3] Bassil F, Brown HJ, Pattabhiraman S, Iwasyk JE, Maghames CM, Meymand ES, Cox TO, Riddle DM, Zhang B, Trojanowski JQ, Lee VM (2020). Amyloid-beta (abeta) plaques promote seeding and spreading of alpha-synuclein and tau in a mouse model of lewy body disorders with abeta pathology. Neuron.

[4] Benitez BA, Davis AA, Jin SC, Ibanez L, Ortega-Cubero S, Pastor P, Choi J, Cooper B, Perlmutter JS, Cruchaga C (2016). Resequencing analysis of five Mendelian genes and the top genes from genome-wide association studies in Parkinson’s disease. Mol Neurodegener.

[5] Blauwendraat C, Heilbron K, Vallerga CL, Bandres-Ciga S, von Coelln R, Pihlstrom L, Simon-Sanchez J, Schulte C, Sharma M, Krohn L, Siitonen A, Iwaki H, Leonard H, Noyce AJ, Tan M, Gibbs JR, Hernandez DG, Scholz SW, Jankovic J, Shulman LM, Lesage S, Corvol JC, Brice A, van Hilten JJ, Marinus J, andMe Research T, Eerola-Rautio J, Tienari P, Majamaa K, Toft M, Grosset DG, Gasser T, Heutink P, Shulman JM, Wood N, Hardy J, Morris HR, Hinds DA, Gratten J, Visscher PM, Gan-Or Z, Nalls MA, Singleton AB, International Parkinson’s Disease Genomics C (2019) Parkinson’s disease age at onset genome-wide association study: defining heritability, genetic loci, and alpha-synuclein mechanisms. Mov Disord 34:866–875. 10.1002/mds.27659 10.1002/mds.27659 PMC6579628 30957308

[6] Bowden J, Del Greco MF, Minelli C, Davey Smith G, Sheehan NA, Thompson JR (2016). Assessing the suitability of summary data for two-sample Mendelian randomization analyses using MR-Egger regression: the role of the I2 statistic. Int J Epidemiol.

[7] Braak H, Braak E (1991). Neuropathological stageing of Alzheimer-related changes. Acta Neuropathol.

[8] Brunnstrom H, Hansson O, Zetterberg H, Londos E, Englund E (2013). Correlations of CSF tau and amyloid levels with Alzheimer pathology in neuropathologically verified dementia with Lewy bodies. Int J Geriatr Psychiatry.

[9] Buddhala C, Campbell MC, Perlmutter JS, Kotzbauer PT (2015). Correlation between decreased CSF alpha-synuclein and Abeta(1)(-)(4)(2) in Parkinson disease. Neurobiol Aging.

[10] Burgess S, Foley CN, Allara E, Staley JR, Howson JMM (2020). A robust and efficient method for Mendelian randomization with hundreds of genetic variants. Nat Commun.

[11] Burgess S, Thompson SG (2017). Interpreting findings from Mendelian randomization using the MR-Egger method. Eur J Epidemiol.

[12] Burgess S, Zuber V, Gkatzionis A, Foley CN (2018). Modal-based estimation via heterogeneity-penalized weighting: model averaging for consistent and efficient estimation in Mendelian randomization when a plurality of candidate instruments are valid. Int J Epidemiol.

[13] Campbell MC, Jackson JJ, Koller JM, Snyder AZ, Kotzbauer PT, Perlmutter JS (2020). Proteinopathy and longitudinal changes in functional connectivity networks in Parkinson disease. Neurology.

[14] Campbell MC, Koller JM, Snyder AZ, Buddhala C, Kotzbauer PT, Perlmutter JS (2015). CSF proteins and resting-state functional connectivity in Parkinson disease. Neurology.

[15] Campbell MC, Myers PS, Weigand AJ, Foster ER, Cairns NJ, Jackson JJ, Lessov-Schlaggar CN, Perlmutter JS (2020). Parkinson disease clinical subtypes: key features & clinical milestones. Ann Clin Transl Neurol.

[16] Chang CC, Chow CC, Tellier LC, Vattikuti S, Purcell SM, Lee JJ (2015). Second-generation PLINK: rising to the challenge of larger and richer datasets. GigaScience.

[17] Choi SW, O’Reilly PF (2019). PRSice-2: polygenic Risk Score software for biobank-scale data. GigaScience.

[18] Consensus recommendations for the postmortem diagnosis of Alzheimer’s disease (1997). The National Institute on Aging, and Reagan Institute Working Group on diagnostic criteria for the neuropathological assessment of Alzheimer’s disease. Neurobiol Aging.

[19] Cruchaga C, Del-Aguila JL, Saef B, Black K, Fernandez MV, Budde J, Ibanez L, Kapoor M, Tosto G, Mayeux RP, Holtzman DM, Fagan AM, Morris JC, Bateman RJ, Goate AM, Harari O, Dominantly Inherited Alzheimer N, Disease Neuroimaging I, study N-Lf (2017). Polygenic risk score of sporadic late-onset Alzheimer’s disease reveals a shared architecture with the familial and early-onset forms. Alzheimer’s Dement J Alzheimer’s Assoc.

[20] Davies NM, Holmes MV, Davey Smith G (2018). Reading Mendelian randomisation studies: a guide, glossary, and checklist for clinicians. BMJ.

[21] Davis AA, Andruska KM, Benitez BA, Racette BA, Perlmutter JS, Cruchaga C (2016). Variants in GBA, SNCA, and MAPT influence Parkinson disease risk, age at onset, and progression. Neurobiol Aging.

[22] Davis AA, Inman CE, Wargel ZM, Dube U, Freeberg BM, Galluppi A, Haines JN, Dhavale DD, Miller R, Choudhury FA, Sullivan PM, Cruchaga C, Perlmutter JS, Ulrich JD, Benitez BA, Kotzbauer PT, Holtzman DM (2020). APOE genotype regulates pathology and disease progression in synucleinopathy. Sci Transl Med.

[23] Delaneau O, Coulonges C, Zagury JF (2008). Shape-IT: new rapid and accurate algorithm for haplotype inference. BMC Bioinform.

[24] Deming Y, Li Z, Kapoor M, Harari O, Del-Aguila JL, Black K, Carrell D, Cai Y, Fernandez MV, Budde J, Ma S, Saef B, Howells B, Huang KL, Bertelsen S, Fagan AM, Holtzman DM, Morris JC, Kim S, Saykin AJ, De Jager PL, Albert M, Moghekar A, O’Brien R, Riemenschneider M, Petersen RC, Blennow K, Zetterberg H, Minthon L, Van Deerlin VM, Lee VM, Shaw LM, Trojanowski JQ, Schellenberg G, Haines JL, Mayeux R, Pericak-Vance MA, Farrer LA, Peskind ER, Li G, Di Narzo AF, Alzheimer’s Disease Neuroimaging I, Alzheimer Disease Genetic C, Kauwe JS, Goate AM, Cruchaga C (2017). Genome-wide association study identifies four novel loci associated with Alzheimer’s endophenotypes and disease modifiers. Acta Neuropathol.

[25] Deming Y, Li Z, Kapoor M, Harari O, Del-Aguila JL, Black K, Carrell D, Cai Y, Fernandez MV, Budde J, Ma S, Saef B, Howells B, Huang KL, Bertelsen S, Fagan AM, Holtzman DM, Morris JC, Kim S, Saykin AJ, De Jager PL, Albert M, Moghekar A, O’Brien R, Riemenschneider M, Petersen RC, Blennow K, Zetterberg H, Minthon L, Van Deerlin VM, Lee VM, Shaw LM, Trojanowski JQ, Schellenberg G, Haines JL, Mayeux R, Pericak-Vance MA, Farrer LA, Peskind ER, Li G, Di Narzo AF, Alzheimer’s Disease Neuroimaging I, Alzheimer Disease Genetic C, Kauwe JS, Goate AM, Cruchaga C (2017). Genome-wide association study identifies four novel loci associated with Alzheimer’s endophenotypes and disease modifiers. Acta Neuropathol.

[26] Dhavale DD, Tsai C, Bagchi DP, Engel LA, Sarezky J, Kotzbauer PT (2017). A sensitive assay reveals structural requirements for alpha-synuclein fibril growth. J Biol Chem.

[27] Dickson DW, Heckman MG, Murray ME, Soto AI, Walton RL, Diehl NN, van Gerpen JA, Uitti RJ, Wszolek ZK, Ertekin-Taner N, Knopman DS, Petersen RC, Graff-Radford NR, Boeve BF, Bu G, Ferman TJ, Ross OA (2018). APOE epsilon4 is associated with severity of Lewy body pathology independent of Alzheimer pathology. Neurology.

[28] Diez-Fairen M, Benitez BA, Ortega-Cubero S, Lorenzo-Betancor O, Cruchaga C, Lorenzo E, Samaranch L, Carcel M, Obeso JA, Rodriguez-Oroz MC, Aguilar M, Coria F, Pastor MA, Pastor P (2018). Pooled-DNA target sequencing of Parkinson genes reveals novel phenotypic associations in Spanish population. Neurobiol Aging.

[29] Dorsey ER, Sherer T, Okun MS, Bloem BR (2018). The emerging evidence of the Parkinson pandemic. J Parkinson’s Dis.

[30] Eriksen JL, Przedborski S, Petrucelli L (2005). Gene dosage and pathogenesis of Parkinson’s disease. Trends Mol Med.

[31] Espay AJ, Kalia LV, Gan-Or Z, Williams-Gray CH, Bedard PL, Rowe SM, Morgante F, Fasano A, Stecher B, Kauffman MA, Farrer MJ, Coffey CS, Schwarzschild MA, Sherer T, Postuma RB, Strafella AP, Singleton AB, Barker RA, Kieburtz K, Olanow CW, Lozano A, Kordower JH, Cedarbaum JM, Brundin P, Standaert DG, Lang AE (2020). Disease modification and biomarker development in Parkinson disease: revision or reconstruction?. Neurology.

[32] Espay AJ, Vizcarra JA, Marsili L, Lang AE, Simon DK, Merola A, Josephs KA, Fasano A, Morgante F, Savica R, Greenamyre JT, Cambi F, Yamasaki TR, Tanner CM, Gan-Or Z, Litvan I, Mata IF, Zabetian CP, Brundin P, Fernandez HH, Standaert DG, Kauffman MA, Schwarzschild MA, Sardi SP, Sherer T, Perry G, Leverenz JB (2019). Revisiting protein aggregation as pathogenic in sporadic Parkinson and Alzheimer diseases. Neurology.

[33] Eusebi P, Giannandrea D, Biscetti L, Abraha I, Chiasserini D, Orso M, Calabresi P, Parnetti L (2017). Diagnostic utility of cerebrospinal fluid alpha-synuclein in Parkinson’s disease: a systematic review and meta-analysis. Mov Disord.

[34] Foster ER, Campbell MC, Burack MA, Hartlein J, Flores HP, Cairns NJ, Hershey T, Perlmutter JS (2010). Amyloid imaging of Lewy body-associated disorders. Mov Disord.

[35] Gao L, Tang H, Nie K, Wang L, Zhao J, Gan R, Huang J, Zhu R, Feng S, Duan Z, Zhang Y, Wang L (2015). Cerebrospinal fluid alpha-synuclein as a biomarker for Parkinson’s disease diagnosis: a systematic review and meta-analysis. Int J Neurosci.

[36] Hemani G, Zheng J, Elsworth B, Wade KH, Haberland V, Baird D, Laurin C, Burgess S, Bowden J, Langdon R, Tan VY, Yarmolinsky J, Shihab HA, Timpson NJ, Evans DM, Relton C, Martin RM, Davey Smith G, Gaunt TR, Haycock PC (2018). The MR-Base platform supports systematic causal inference across the human phenome. eLife.

[37] Hinrichs AL, Mintun MA, Head D, Fagan AM, Holtzman DM, Morris JC, Goate AM (2010). Cortical binding of pittsburgh compound B, an endophenotype for genetic studies of Alzheimer’s disease. Biol Psychiatry.

[38] Howie B, Marchini J, Stephens M (2011). Genotype imputation with thousands of genomes.

[39] Hughes AJ, Daniel SE, Kilford L, Lees AJ (1992). Accuracy of clinical diagnosis of idiopathic Parkinson’s disease: a clinico-pathological study of 100 cases. J Neurol Neurosurg Psychiatry.

[40] Ibanez L, Dube U, Davis AA, Fernandez MV, Budde J, Cooper B, Diez-Fairen M, Ortega-Cubero S, Pastor P, Perlmutter JS, Cruchaga C, Benitez BA (2018). Pleiotropic effects of variants in dementia genes in Parkinson disease. Front Neurosci.

[41] Ibanez L, Dube U, Saef B, Budde J, Black K, Medvedeva A, Del-Aguila JL, Davis AA, Perlmutter JS, Harari O, Benitez BA, Cruchaga C (2017). Parkinson disease polygenic risk score is associated with Parkinson disease status and age at onset but not with alpha-synuclein cerebrospinal fluid levels. BMC Neurol.

[42] Ibanez L, Heitsch L, Dube U, Farias FHG, Budde J, Bergmann K, Davenport R, Bradley J, Carrera C, Kinnunen J, Sallinen H, Strbian D, Slowik A, Fernandez-Cadenas I, Montaner J, Lee JM, Cruchaga C (2019). Overlap in the genetic architecture of stroke risk, early neurological changes, and cardiovascular risk factors. Stroke.

[43] Irwin DJ, Grossman M, Weintraub D, Hurtig HI, Duda JE, Xie SX, Lee EB, Van Deerlin VM, Lopez OL, Kofler JK, Nelson PT, Jicha GA, Woltjer R, Quinn JF, Kaye J, Leverenz JB, Tsuang D, Longfellow K, Yearout D, Kukull W, Keene CD, Montine TJ, Zabetian CP, Trojanowski JQ (2017). Neuropathological and genetic correlates of survival and dementia onset in synucleinopathies: a retrospective analysis. Lancet Neurol.

[44] Irwin DJ, White MT, Toledo JB, Xie SX, Robinson JL, Van Deerlin V, Lee VM, Leverenz JB, Montine TJ, Duda JE, Hurtig HI, Trojanowski JQ (2012). Neuropathologic substrates of Parkinson disease dementia. Ann Neurol.

[45] Kang JH, Irwin DJ, Chen-Plotkin AS, Siderowf A, Caspell C, Coffey CS, Waligorska T, Taylor P, Pan S, Frasier M, Marek K, Kieburtz K, Jennings D, Simuni T, Tanner CM, Singleton A, Toga AW, Chowdhury S, Mollenhauer B, Trojanowski JQ, Shaw LM, Parkinson’s Progression Markers I (2013). Association of cerebrospinal fluid beta-amyloid 1-42, T-tau, P-tau181, and alpha-synuclein levels with clinical features of drug-naive patients with early Parkinson disease. JAMA Neurol.

[46] Khachaturian ZS (1985). Diagnosis of Alzheimer’s disease. Arch Neurol.

[47] Kotzbauer PT, Cairns NJ, Campbell MC, Willis AW, Racette BA, Tabbal SD, Perlmutter JS (2012). Pathologic accumulation of alpha-synuclein and Abeta in Parkinson disease patients with dementia. Arch Neurol.

[48] Kunkle BW, Grenier-Boley B, Sims R, Bis JC, Damotte V, Naj AC, Boland A, Vronskaya M, van der Lee SJ, Amlie-Wolf A, Bellenguez C, Frizatti A, Chouraki V, Martin ER, Sleegers K, Badarinarayan N, Jakobsdottir J, Hamilton-Nelson KL, Moreno-Grau S, Olaso R, Raybould R, Chen Y, Kuzma AB, Hiltunen M, Morgan T, Ahmad S, Vardarajan BN, Epelbaum J, Hoffmann P, Boada M, Beecham GW, Garnier JG, Harold D, Fitzpatrick AL, Valladares O, Moutet ML, Gerrish A, Smith AV, Qu L, Bacq D, Denning N, Jian X, Zhao Y, Del Zompo M, Fox NC, Choi SH, Mateo I, Hughes JT, Adams HH, Malamon J, Sanchez-Garcia F, Patel Y, Brody JA, Dombroski BA, Naranjo MCD, Daniilidou M, Eiriksdottir G, Mukherjee S, Wallon D, Uphill J, Aspelund T, Cantwell LB, Garzia F, Galimberti D, Hofer E, Butkiewicz M, Fin B, Scarpini E, Sarnowski C, Bush WS, Meslage S, Kornhuber J, White CC, Song Y, Barber RC, Engelborghs S, Sordon S, Voijnovic D, Adams PM, Vandenberghe R, Mayhaus M, Cupples LA, Albert MS, De Deyn PP, Gu W, Himali JJ, Beekly D, Squassina A, Hartmann AM, Orellana A, Blacker D, Rodriguez-Rodriguez E, Lovestone S, Garcia ME, Doody RS, Munoz-Fernadez C, Sussams R, Lin H, Fairchild TJ, Benito YA, Holmes C, Karamujic-Comic H, Frosch MP, Thonberg H, Maier W, Roshchupkin G, Ghetti B, Giedraitis V, Kawalia A, Li S, Huebinger RM, Kilander L, Moebus S, Hernandez I, Kamboh MI, Brundin R, Turton J, Yang Q, Katz MJ, Concari L, Lord J, Beiser AS, Keene CD, Helisalmi S, Kloszewska I, Kukull WA, Koivisto AM, Lynch A, Tarraga L, Larson EB, Haapasalo A, Lawlor B, Mosley TH, Lipton RB, Solfrizzi V, Gill M, Longstreth WT, Jr., Montine TJ, Frisardi V, Diez-Fairen M, Rivadeneira F, Petersen RC, Deramecourt V, Alvarez I, Salani F, Ciaramella A, Boerwinkle E, Reiman EM, Fievet N, Rotter JI, Reisch JS, Hanon O, Cupidi C, Andre Uitterlinden AG, Royall DR, Dufouil C, Maletta RG, de Rojas I, Sano M, Brice A, Cecchetti R, George-Hyslop PS, Ritchie K, Tsolaki M, Tsuang DW, Dubois B, Craig D, Wu CK, Soininen H, Avramidou D, Albin RL, Fratiglioni L, Germanou A, Apostolova LG, Keller L, Koutroumani M, Arnold SE, Panza F, Gkatzima O, Asthana S, Hannequin D, Whitehead P, Atwood CS, Caffarra P, Hampel H, Quintela I, Carracedo A, Lannfelt L, Rubinsztein DC, Barnes LL, Pasquier F, Frolich L, Barral S, McGuinness B, Beach TG, Johnston JA, Becker JT, Passmore P, Bigio EH, Schott JM, Bird TD, Warren JD, Boeve BF, Lupton MK, Bowen JD, Proitsi P, Boxer A, Powell JF, Burke JR, Kauwe JSK, Burns JM, Mancuso M, Buxbaum JD, Bonuccelli U, Cairns NJ, McQuillin A, Cao C, Livingston G, Carlson CS, Bass NJ, Carlsson CM, Hardy J, Carney RM, Bras J, Carrasquillo MM, Guerreiro R, Allen M, Chui HC, Fisher E, Masullo C, Crocco EA, DeCarli C, Bisceglio G, Dick M, Ma L, Duara R, Graff-Radford NR, Evans DA, Hodges A, Faber KM, Scherer M, Fallon KB, Riemenschneider M, Fardo DW, Heun R, Farlow MR, Kolsch H, Ferris S, Leber M, Foroud TM, Heuser I, Galasko DR, Giegling I, Gearing M, Hull M, Geschwind DH, Gilbert JR, Morris J, Green RC, Mayo K, Growdon JH, Feulner T, Hamilton RL, Harrell LE, Drichel D, Honig LS, Cushion TD, Huentelman MJ, Hollingworth P, Hulette CM, Hyman BT, Marshall R, Jarvik GP, Meggy A, Abner E, Menzies GE, Jin LW, Leonenko G, Real LM, Jun GR, Baldwin CT, Grozeva D, Karydas A, Russo G, Kaye JA, Kim R, Jessen F, Kowall NW, Vellas B, Kramer JH, Vardy E, LaFerla FM, Jockel KH, Lah JJ, Dichgans M, Leverenz JB, Mann D, Levey AI, Pickering-Brown S, Lieberman AP, Klopp N, Lunetta KL, Wichmann HE, Lyketsos CG, Morgan K, Marson DC, Brown K, Martiniuk F, Medway C, Mash DC, Nothen MM, Masliah E, Hooper NM, McCormick WC, Daniele A, McCurry SM, Bayer A, McDavid AN, Gallacher J, McKee AC, van den Bussche H, Mesulam M, Brayne C, Miller BL, Riedel-Heller S, Miller CA, Miller JW, Al-Chalabi A, Morris JC, Shaw CE, Myers AJ, Wiltfang J, O’Bryant S, Olichney JM, Alvarez V, Parisi JE, Singleton AB, Paulson HL, Collinge J, Perry WR, Mead S, Peskind E, Cribbs DH, Rossor M, Pierce A, Ryan NS, Poon WW, Nacmias B, Potter H, Sorbi S, Quinn JF, Sacchinelli E, Raj A, Spalletta G, Raskind M, Caltagirone C, Bossu P, Orfei MD, Reisberg B, Clarke R, Reitz C, Smith AD, Ringman JM, Warden D, Roberson ED, Wilcock G, Rogaeva E, Bruni AC, Rosen HJ, Gallo M, Rosenberg RN, Ben-Shlomo Y, Sager MA, Mecocci P, Saykin AJ, Pastor P, Cuccaro ML, Vance JM, Schneider JA, Schneider LS, Slifer S, Seeley WW, Smith AG, Sonnen JA, Spina S, Stern RA, Swerdlow RH, Tang M, Tanzi RE, Trojanowski JQ, Troncoso JC, Van Deerlin VM, Van Eldik LJ, Vinters HV, Vonsattel JP, Weintraub S, Welsh-Bohmer KA, Wilhelmsen KC, Williamson J, Wingo TS, Woltjer RL, Wright CB, Yu CE, Yu L, Saba Y, Pilotto A, Bullido MJ, Peters O, Crane PK, Bennett D, Bosco P, Coto E, Boccardi V, De Jager PL, Lleo A, Warner N, Lopez OL, Ingelsson M, Deloukas P, Cruchaga C, Graff C, Gwilliam R, Fornage M, Goate AM, Sanchez-Juan P, Kehoe PG, Amin N, Ertekin-Taner N, Berr C, Debette S, Love S, Launer LJ, Younkin SG, Dartigues JF, Corcoran C, Ikram MA, Dickson DW, Nicolas G, Campion D, Tschanz J, Schmidt H, Hakonarson H, Clarimon J, Munger R, Schmidt R, Farrer LA, Van Broeckhoven C, M COD, DeStefano AL, Jones L, Haines JL, Deleuze JF, Owen MJ, Gudnason V, Mayeux R, Escott-Price V, Psaty BM, Ramirez A, Wang LS, Ruiz A, van Duijn CM, Holmans PA, Seshadri S, Williams J, Amouyel P, Schellenberg GD, Lambert JC, Pericak-Vance MA, Alzheimer Disease Genetics C, European Alzheimer’s Disease I, Cohorts for H, Aging Research in Genomic Epidemiology C, Genetic, Environmental Risk in Ad/Defining Genetic P, Environmental Risk for Alzheimer’s Disease C (2019) Genetic meta-analysis of diagnosed Alzheimer’s disease identifies new risk loci and implicates Abeta, tau, immunity and lipid processing. Nat Genet 51:414–430. 10.1038/s41588-019-0358-2 10.1038/s41588-019-0358-2 PMC6463297 30820047

[49] Logan J, Fowler JS, Volkow ND, Wang GJ, Ding YS, Alexoff DL (1996). Distribution volume ratios without blood sampling from graphical analysis of PET data. J Cerebral Blood Flow Metab.

[50] Mintun MA, Larossa GN, Sheline YI, Dence CS, Lee SY, Mach RH, Klunk WE, Mathis CA, DeKosky ST, Morris JC (2006). [11C]PIB in a nondemented population: potential antecedent marker of Alzheimer disease. Neurology.

[51] Mirra SS, Heyman A, McKeel D, Sumi SM, Crain BJ, Brownlee LM, Vogel FS, Hughes JP, van Belle G, Berg L (1991). The Consortium to Establish a Registry for Alzheimer’s Disease (CERAD). Part II. Standardization of the neuropathologic assessment of Alzheimer’s disease. Neurology.

[52] Mollenhauer B, Caspell-Garcia CJ, Coffey CS, Taylor P, Shaw LM, Trojanowski JQ, Singleton A, Frasier M, Marek K, Galasko D, Parkinson’s Progression Marker I (2017). Longitudinal CSF biomarkers in patients with early Parkinson disease and healthy controls. Neurology.

[53] Mollenhauer B, Zimmermann J, Sixel-Doring F, Focke NK, Wicke T, Ebentheuer J, Schaumburg M, Lang E, Friede T, Trenkwalder C, DeNoPa Study G (2019). Baseline predictors for progression 4 years after Parkinson’s disease diagnosis in the De Novo Parkinson Cohort (DeNoPa). Mov Disord.

[54] Nalls MA, Blauwendraat C, Vallerga CL, Heilbron K, Bandres-Ciga S, Chang D, Tan M, Kia DA, Noyce AJ, Xue A, Bras J, Young E, von Coelln R, Simon-Sanchez J, Schulte C, Sharma M, Krohn L, Pihlstrom L, Siitonen A, Iwaki H, Leonard H, Faghri F, Gibbs JR, Hernandez DG, Scholz SW, Botia JA, Martinez M, Corvol JC, Lesage S, Jankovic J, Shulman LM, Sutherland M, Tienari P, Majamaa K, Toft M, Andreassen OA, Bangale T, Brice A, Yang J, Gan-Or Z, Gasser T, Heutink P, Shulman JM, Wood NW, Hinds DA, Hardy JA, Morris HR, Gratten J, Visscher PM, Graham RR, Singleton AB, andMe Research T, System Genomics of Parkinson’s Disease C, International Parkinson’s Disease Genomics C (2019) Identification of novel risk loci, causal insights, and heritable risk for Parkinson’s disease: a meta-analysis of genome-wide association studies. Lancet Neurol 18:1091–1102. 10.1016/S1474-4422(19)30320-5 10.1016/S1474-4422(19)30320-5 PMC8422160 31701892

[55] Polymeropoulos MH, Lavedan C, Leroy E, Ide SE, Dehejia A, Dutra A, Pike B, Root H, Rubenstein J, Boyer R, Stenroos ES, Chandrasekharappa S, Athanassiadou A, Papapetropoulos T, Johnson WG, Lazzarini AM, Duvoisin RC, Di Iorio G, Golbe LI, Nussbaum RL (1997). Mutation in the alpha-synuclein gene identified in families with Parkinson’s disease. Science.

[56] Purcell S, Neale B, Todd-Brown K, Thomas L, Ferreira MA, Bender D, Maller J, Sklar P, de Bakker PI, Daly MJ, Sham PC (2007). PLINK: a tool set for whole-genome association and population-based linkage analyses. Am J Hum Genet.

[57] Rizek P, Kumar N, Jog MS (2016). An update on the diagnosis and treatment of Parkinson disease. CMAJ Can Med Assoc J journal de l’Association medicale canadienne.

[58] Robinson JL, Lee EB, Xie SX, Rennert L, Suh E, Bredenberg C, Caswell C, Van Deerlin VM, Yan N, Yousef A, Hurtig HI, Siderowf A, Grossman M, McMillan CT, Miller B, Duda JE, Irwin DJ, Wolk D, Elman L, McCluskey L, Chen-Plotkin A, Weintraub D, Arnold SE, Brettschneider J, Lee VM, Trojanowski JQ (2018). Neurodegenerative disease concomitant proteinopathies are prevalent, age-related and APOE4-associated. Brain J Neurol.

[59] Rongve A, Witoelar A, Ruiz A, Athanasiu L, Abdelnour C, Clarimon J, Heilmann-Heimbach S, Hernandez I, Moreno-Grau S, de Rojas I, Morenas-Rodriguez E, Fladby T, Sando SB, Brathen G, Blanc F, Bousiges O, Lemstra AW, van Steenoven I, Londos E, Almdahl IS, Palhaugen L, Eriksen JA, Djurovic S, Stordal E, Saltvedt I, Ulstein ID, Bettella F, Desikan RS, Idland AV, Toft M, Pihlstrom L, Snaedal J, Tarraga L, Boada M, Lleo A, Stefansson H, Stefansson K, Ramirez A, Aarsland D, Andreassen OA (2019). Author Correction: GBA and APOE epsilon4 associate with sporadic dementia with Lewy bodies in European genome wide association study. Sci Rep.

[60] Rongve A, Witoelar A, Ruiz A, Athanasiu L, Abdelnour C, Clarimon J, Heilmann-Heimbach S, Hernandez I, Moreno-Grau S, de Rojas I, Morenas-Rodriguez E, Fladby T, Sando SB, Brathen G, Blanc F, Bousiges O, Lemstra AW, van Steenoven I, Londos E, Almdahl IS, Palhaugen L, Eriksen JA, Djurovic S, Stordal E, Saltvedt I, Ulstein ID, Bettella F, Desikan RS, Idland AV, Toft M, Pihlstrom L, Snaedal J, Tarraga L, Boada M, Lleo A, Stefansson H, Stefansson K, Ramirez A, Aarsland D, Andreassen OA (2019). GBA and APOE epsilon4 associate with sporadic dementia with Lewy bodies in European genome wide association study. Sci Rep.

[61] Rowland DJ, Garbow JR, Laforest R, Snyder AZ (2005). Registration of [18F]FDG microPET and small-animal MRI. Nucl Med Biol.

[62] Shi Y, Yamada K, Liddelow SA, Smith ST, Zhao L, Luo W, Tsai RM, Spina S, Grinberg LT, Rojas JC, Gallardo G, Wang K, Roh J, Robinson G, Finn MB, Jiang H, Sullivan PM, Baufeld C, Wood MW, Sutphen C, McCue L, Xiong C, Del-Aguila JL, Morris JC, Cruchaga C, Alzheimer’s Disease Neuroimaging I, Fagan AM, Miller BL, Boxer AL, Seeley WW, Butovsky O, Barres BA, Paul SM, Holtzman DM (2017). ApoE4 markedly exacerbates tau-mediated neurodegeneration in a mouse model of tauopathy. Nature.

[63] Siderowf A, Xie SX, Hurtig H, Weintraub D, Duda J, Chen-Plotkin A, Shaw LM, Van Deerlin V, Trojanowski JQ, Clark C (2010). CSF amyloid beta 1–42 predicts cognitive decline in Parkinson disease. Neurology.

[64] Stewart T, Liu C, Ginghina C, Cain KC, Auinger P, Cholerton B, Shi M, Zhang J, Parkinson Study Group DI (2014). Cerebrospinal fluid alpha-synuclein predicts cognitive decline in Parkinson disease progression in the DATATOP cohort. Am J Pathol.

[65] Tsuang D, Leverenz JB, Lopez OL, Hamilton RL, Bennett DA, Schneider JA, Buchman AS, Larson EB, Crane PK, Kaye JA, Kramer P, Woltjer R, Trojanowski JQ, Weintraub D, Chen-Plotkin AS, Irwin DJ, Rick J, Schellenberg GD, Watson GS, Kukull W, Nelson PT, Jicha GA, Neltner JH, Galasko D, Masliah E, Quinn JF, Chung KA, Yearout D, Mata IF, Wan JY, Edwards KL, Montine TJ, Zabetian CP (2013). APOE epsilon4 increases risk for dementia in pure synucleinopathies. JAMA Neurol.

[66] Turley P, Walters RK, Maghzian O, Okbay A, Lee JJ, Fontana MA, Nguyen-Viet TA, Wedow R, Zacher M, Furlotte NA, Magnusson P, Oskarsson S, Johannesson M, Visscher PM, Laibson D, Cesarini D, Neale BM, Benjamin DJ, andMe Research T, Social Science Genetic Association C (2018). Multi-trait analysis of genome-wide association summary statistics using MTAG. Nat Genet.

[67] Twohig D, Rodriguez-Vieitez E, Sando SB, Berge G, Lauridsen C, Moller I, Grontvedt GR, Brathen G, Patra K, Bu G, Benzinger TLS, Karch CM, Fagan A, Morris JC, Bateman RJ, Nordberg A, White LR, Nielsen HM, Dominantly Inherited Alzheimer N (2018). The relevance of cerebrospinal fluid alpha-synuclein levels to sporadic and familial Alzheimer’s disease. Acta Neuropathol Commun.

[68] Vassos E, Di Forti M, Coleman J, Iyegbe C, Prata D, Euesden J, O’Reilly P, Curtis C, Kolliakou A, Patel H, Newhouse S, Traylor M, Ajnakina O, Mondelli V, Marques TR, Gardner-Sood P, Aitchison KJ, Powell J, Atakan Z, Greenwood KE, Smith S, Ismail K, Pariante C, Gaughran F, Dazzan P, Markus HS, David AS, Lewis CM, Murray RM, Breen G (2016). An examination of polygenic score risk prediction in individuals with first-episode psychosis. Biol Psychiatry.

[69] Willer CJ, Li Y, Abecasis GR (2010). METAL: fast and efficient meta-analysis of genomewide association scans. Bioinformatics.

[70] Yang C, Farias F, Ibanez L, Sadler B, Fernandez M-V, Wang F, Bradley J, Eiffert B, Bahena J, Budde J, Li Z, Dube U, Sung YJ, Mihindukulasuriya K, Morris J, Fagan A, Perrin R, Benitez B, Rhinn H, Harari O, Cruchaga C (2020). Genomic and multi-tissue proteomic integration for understanding the biology of disease and other complex traits. medRxiv.

[71] Yang J, Lee SH, Goddard ME, Visscher PM (2011). GCTA: a tool for genome-wide complex trait analysis. Am J Hum Genet.

[72] Yavorska OO, Burgess S (2017). MendelianRandomization: an R package for performing Mendelian randomization analyses using summarized data. Int J Epidemiol.

[73] Zhong XL, Li JQ, Sun L, Li YQ, Wang HF, Cao XP, Tan CC, Wang L, Tan L, Yu JT, Alzheimer’s Disease Neuroimaging I (2019). A genome-wide association study of alpha-synuclein levels in cerebrospinal fluid. Neurotox Res.

