# Supplementary material for: Functional genomic analyses uncover APOE-mediated regulation of brain and cerebrospinal fluid beta-amyloid levels in Parkinson disease
Source: Acta Neuropathol Commun. 2020 Nov 19;8:196. doi: 10.1186/s40478-020-01072-8 (PMC7678051; doi:10.1186/s40478-020-01072-8)

Supplementary Figure 1. Association plots of single variant analyses of CSF Alpha Synuclein by Dataset. Each Manhattan plot shows the negative $\log_{10}$-transformed p-values for the analyses of α-Syn in **A.** PD cohort from WUSTL in which α-Syn was measured using ELISA based methds **B.** the PPMI cohort **C.** the WUSTL cohort in which α-Syn was measured using the SomaScan platform **D.** the ADNI cohort and **E.** the cohort from Spain. The X-axis represent the genomic location. The horizontal lines represent the genome-wide significance threshold, $P = 5 \times 10^{-8}$ (red) and suggestive threshold, $P = 1 \times 10^{-5}$ (blue). Suggestive SNPs for α-Syn, can be found in Table S3

A.

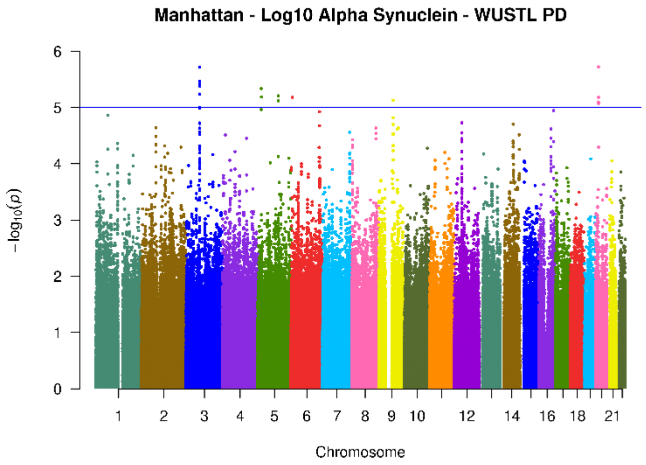

B.

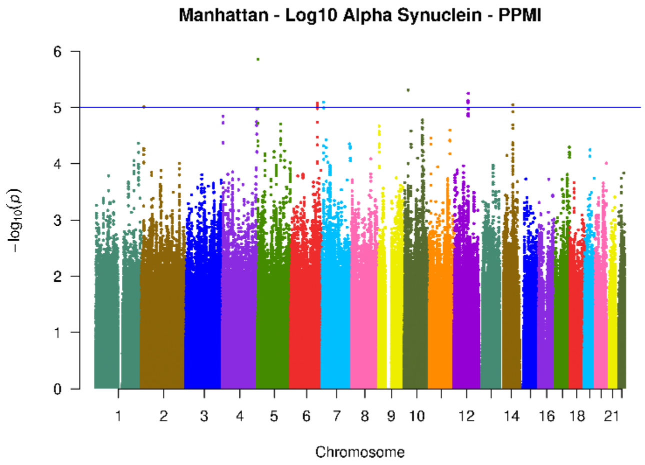

C.

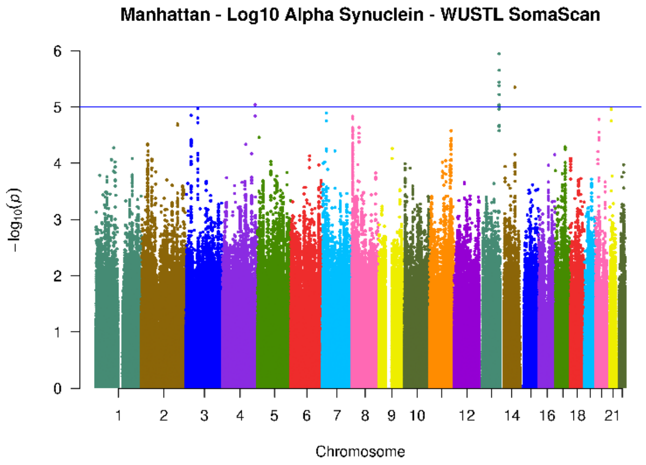

D.

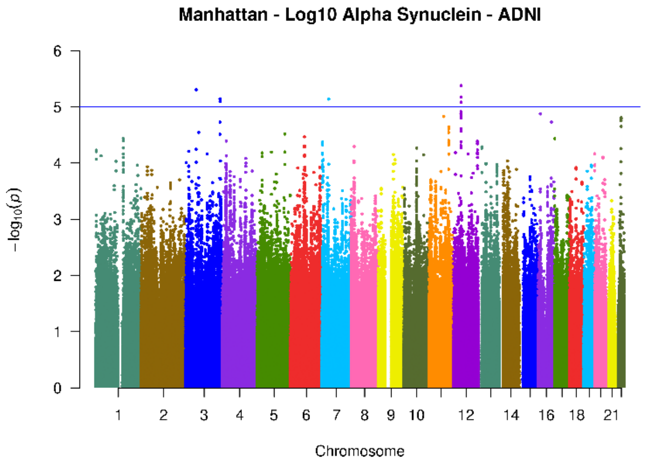

E.

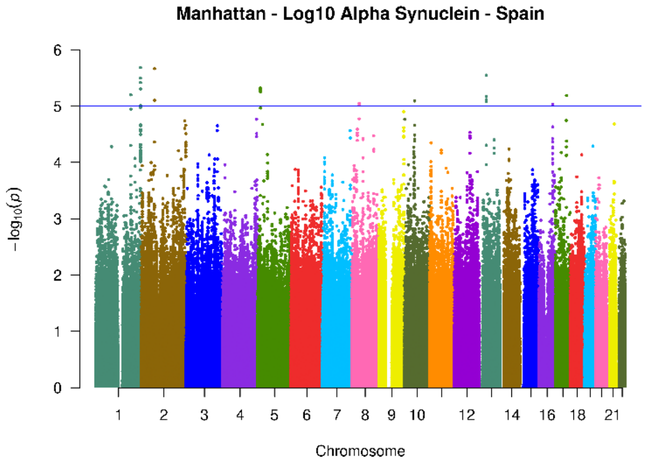

Supplementary Figure 2: Association plots of single variant analyses of Alpha Synuclein in the WUSTL cohort measured with SomaScan. Each Manhattan plot shows the negative log10-transformed p-values fir the analyses of α-Syn in **A.** CSF **B.** Plasma and **C.** Brain. The final panel **D.** shows the results for the Multi-tissue analyses using the software MTAG that removes the individual variation and increase the power to find a non-tissue specific modulator of α-Syn. The X-axis represent the genomic location. The horizontal lines represent the genome-wide significance threshold, $p=5\times10^{-8}$ (red) and suggestive threshold, $p=1\times10^{-5}$ (blue). **E**, **F** Regional association plots for the suggestive loci identified by MTAG are shown for the chr3 locus (**E**) and the chr13 locus (**F**). The SNPs labeled on each regional plot had the lowest p-value at each locus and are represented by a purple diamond. Each dot represents an SNP, and dot colors indicate linkage disequilibrium with the labeled SNP. Blue vertical lines show the recombination rate marked on the right-hand y-axis of each regional plot. Suggestive SNPs for α-Syn using MTAG, can be found in Table S4

A.

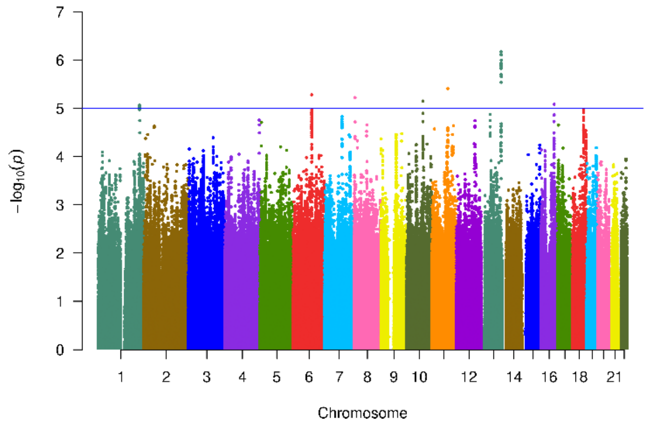

B.

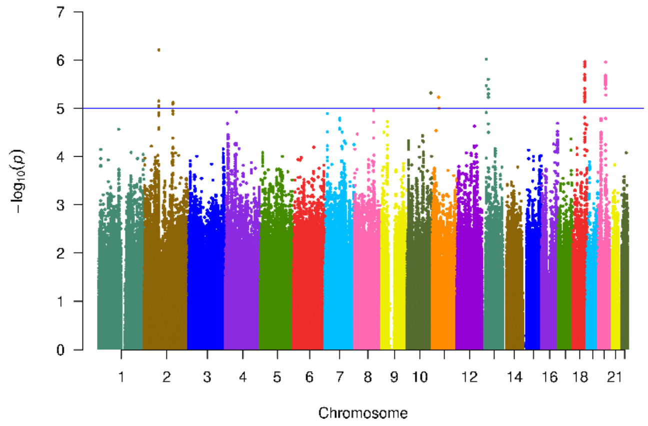

C.

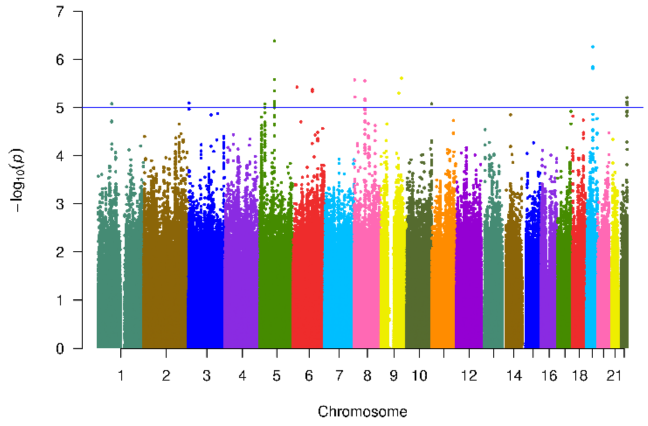

D.

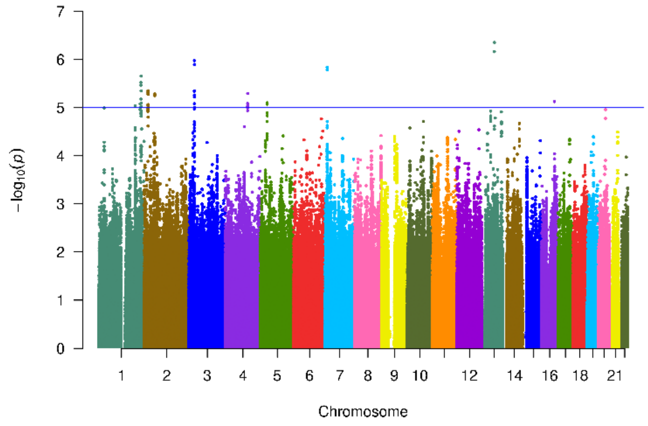

E.

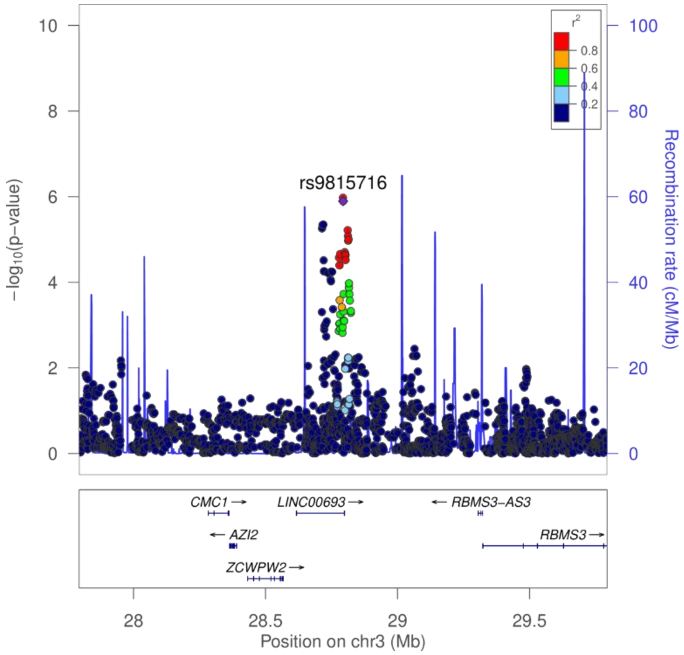

rs9815716
−log10(p−value)
Recombination rate (cM/Mb)
r²
0.8
0.6
0.4
0.2
0
2
4
6
8
10
0
20
40
60
80
100
CMC1
LINC00693
RBMS3−AS3
AZI2
RBMS3
ZCWPW2
28
28.5
29
29.5
Position on chr3 (Mb)

F.

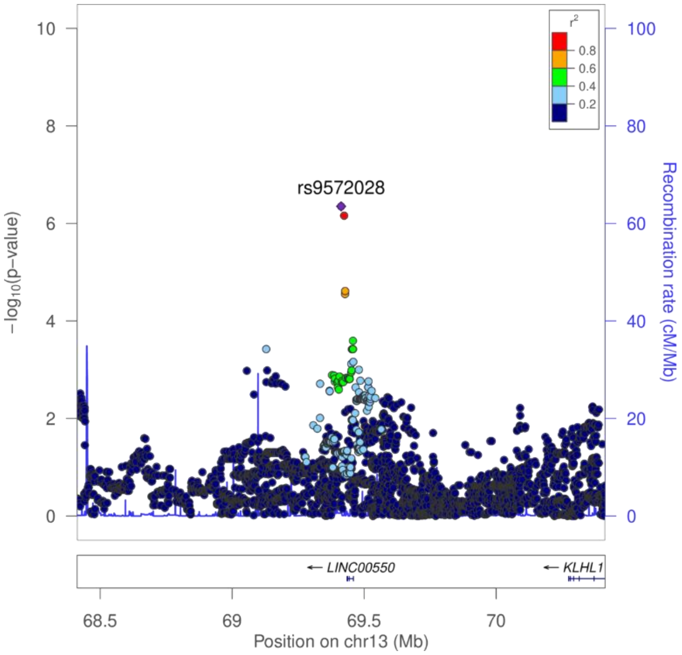

rs9572028
−log10(p−value)
Recombination rate (cM/Mb)
r²
0.8
0.6
0.4
0.2
0
2
4
6
8
10
0
20
40
60
80
100
LINC00550
KLHL1
68.5
69
69.5
70
Position on chr13 (Mb)

Supplementary Figure 3: Association plots of single variant analyses of CSF Total tau by Dataset. Each Manhattan plot shows the negative $\log_{10}$-transformed p-values for the analyses of t-tau in **A.** the WUSTL cohort **B.** the PPMI cohort **C.** the ADNI cohort and **D.** the cohort from Spain. The X-axis represent the genomic location. The horizontal lines represent the genome-wide significance threshold, $p=5\times10^{-8}$ (red) and suggestive threshold, $p=1\times10^{-5}$ (blue). Suggestive SNPs for t-tau, can be found in Table S5

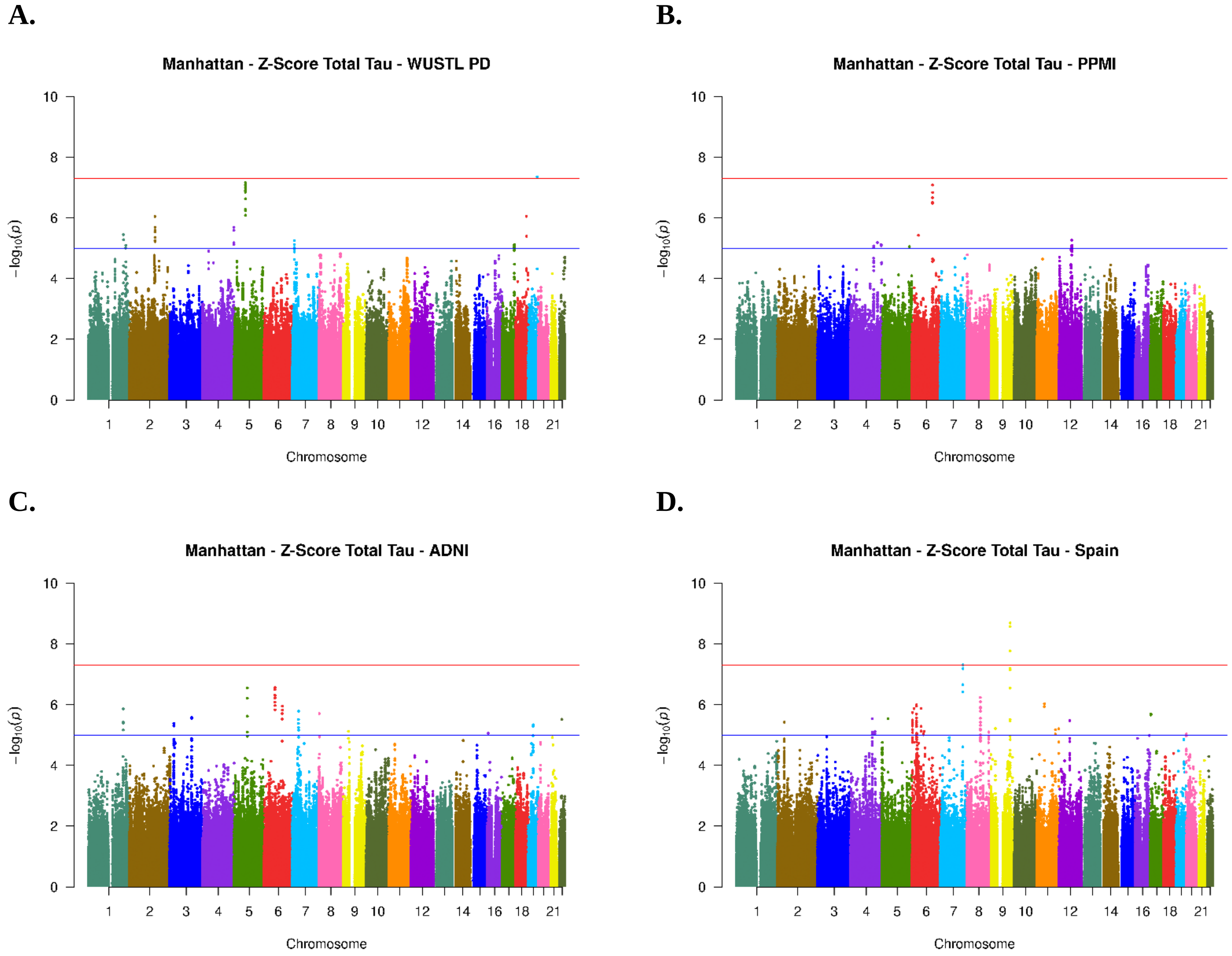

Supplementary Figure 4: Association plots of single variant analyses of CSF Phosphorylated tau by Dataset. Each Manhattan plot shows the negative log_{10}-transformed p-values for the analyses of p-tau_{181} in **A.** the WUSTL cohort **B.** the PPMI cohort **C.** the ADNI cohort and **D.** the cohort from Spain. The X-axis represent the genomic location. The horizontal lines represent the genome-wide significance threshold, $p=5\times10^{-8}$ (red) and suggestive threshold, $p=1\times10^{-5}$ (blue). Suggestive SNPs for p-tau_{181}, can be found in Table S6

A.

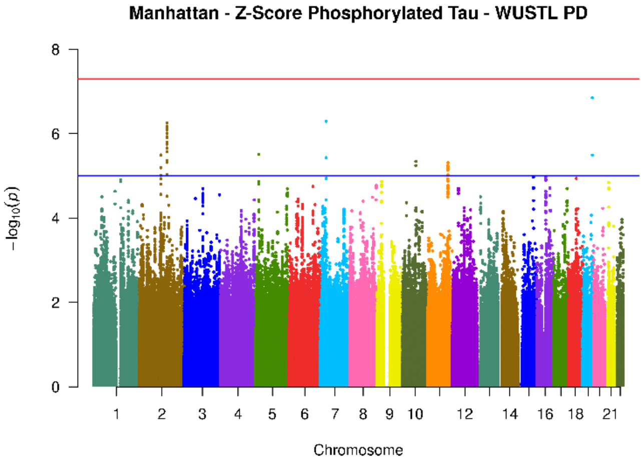

B.

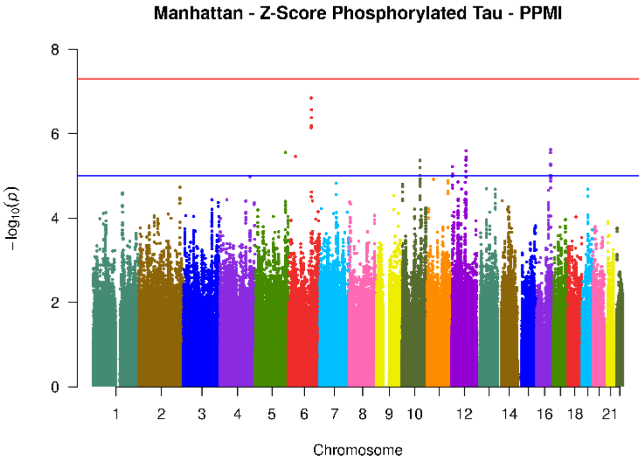

C.

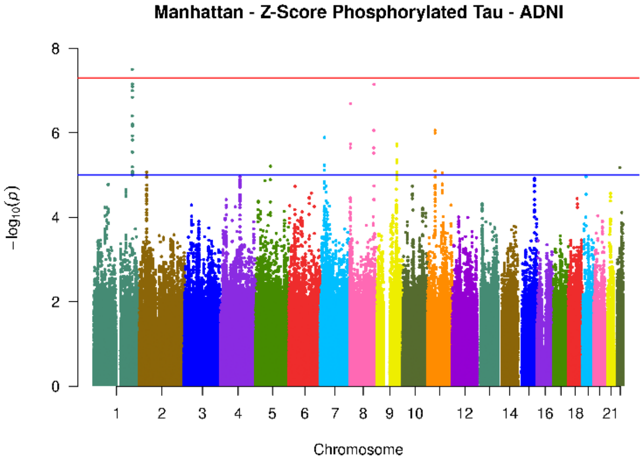

D.

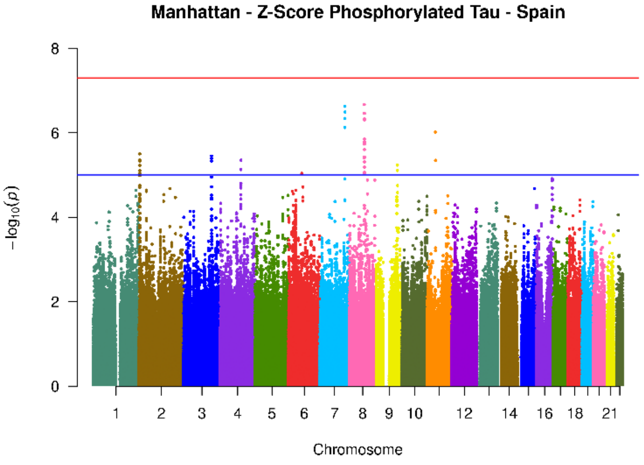

Supplementary Figure 5: Association plots of single variant analyses of CSF Amyloid Beta by Dataset. Each Manhattan plot shows the negative $\log_{10}$-transformed p-values for the analyses of Aβ42 in **A.** the WUSTL cohort **B.** the PPMI cohort **C.** the ADNI cohort and **D.** the cohort from Spain. The X-axis represent the genomic location. The horizontal lines represent the genome-wide significance threshold, $p=5\times10^{-8}$ (red) and suggestive threshold, $p=1\times10^{-5}$ (blue). Suggestive SNPs for p-tau_{181}, can be found in Table S7

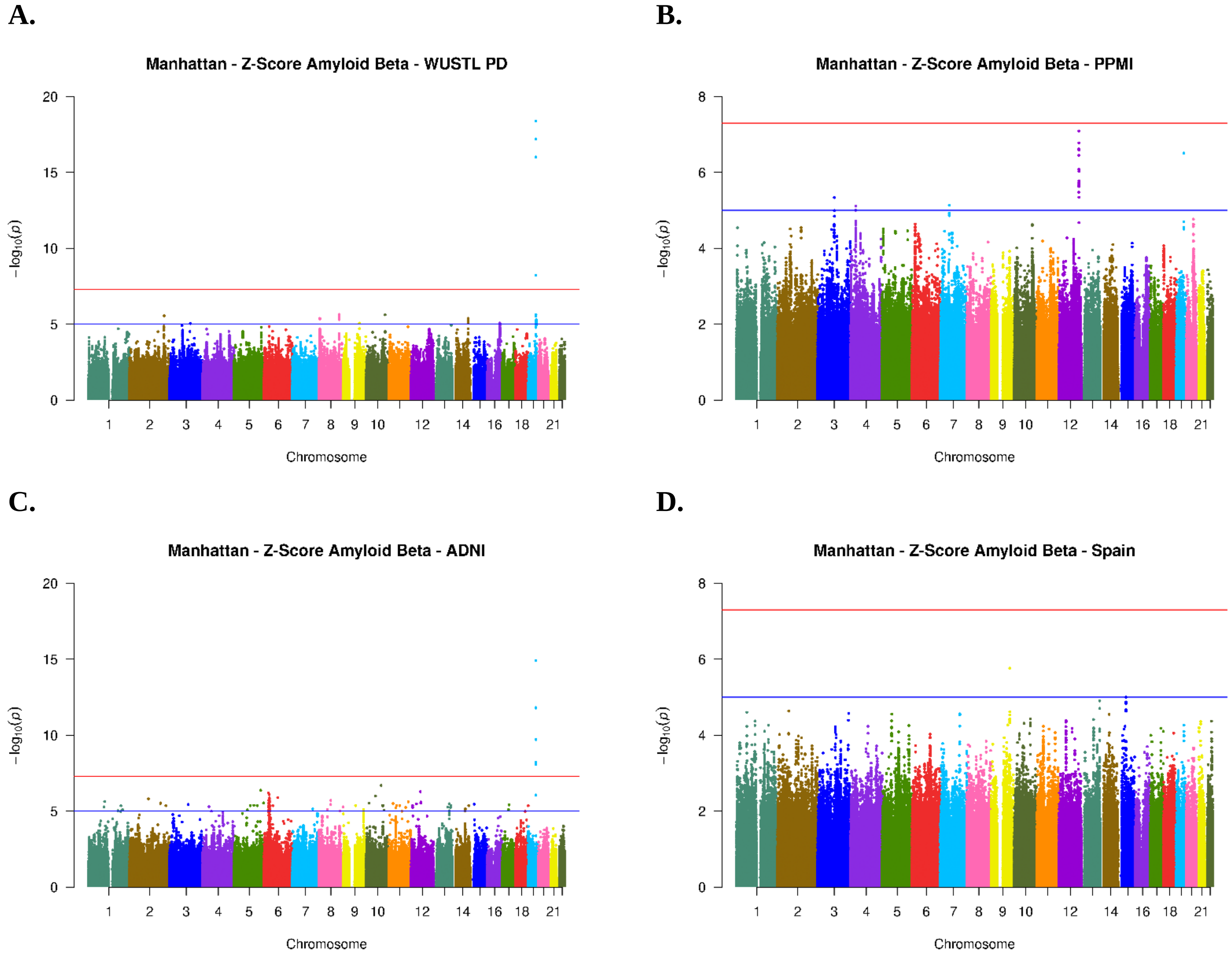

Supplementary Figure 6: Normalized CSF levels of neurodegeneration biomarkers in PD stratified by quartiles (first quartile blue, second green, third gray and forth orange) of polygenic risk scores computed using summary statistics from META-PD

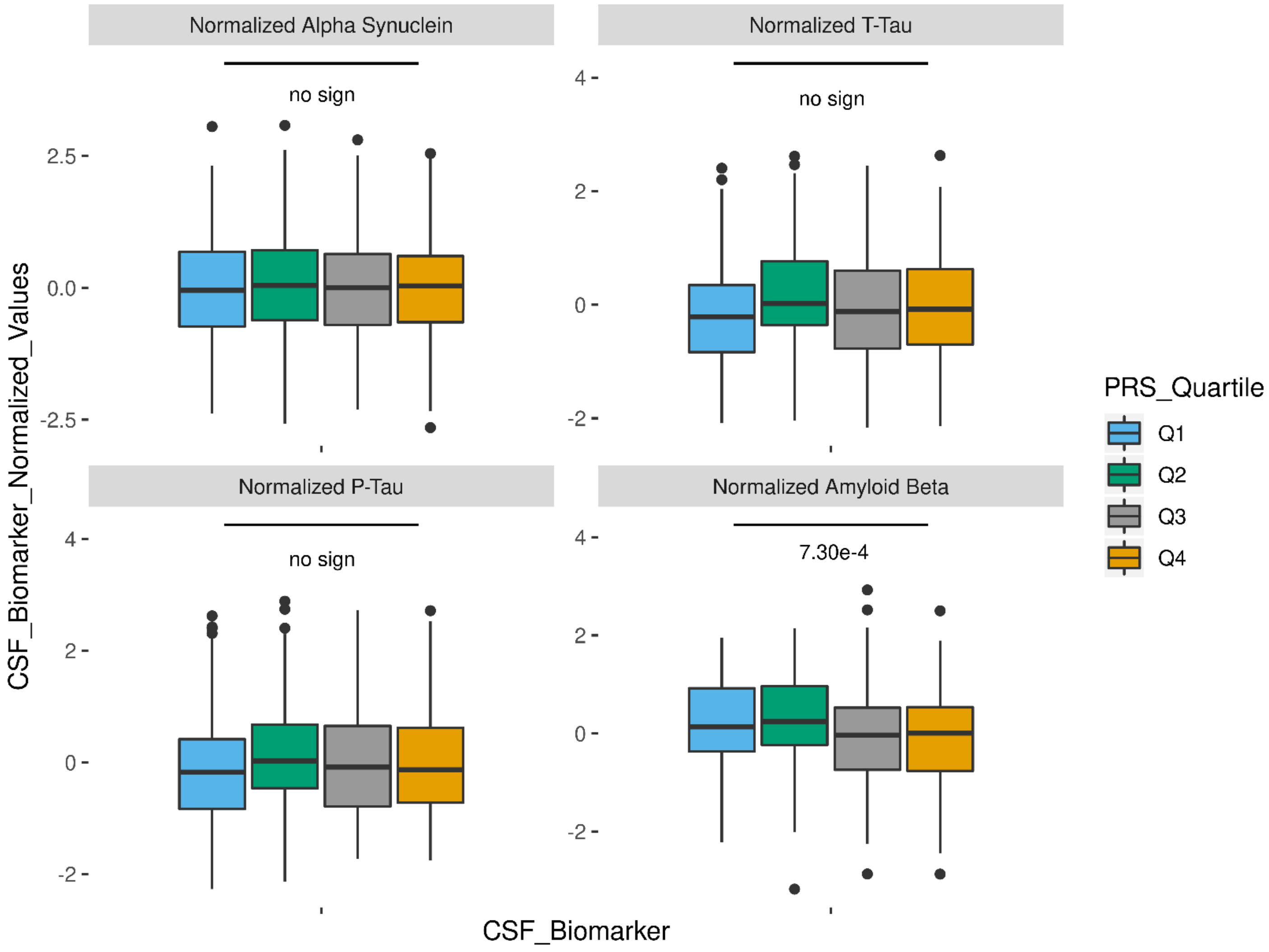

Supplementary Figure 7: Mendelian randomization analyses. Regression of all methods and leave-one-out analyses for Alpha Synuclein (**A** and **B**), and the regression for all methods for Total Tau (**C**) and Phosphorylated Tau (**D**). Regression plots show the linear regression using each of the MR methods tested (color-coded). X and Y axis represent the genetic association of the two tested datasets (outcome and exposure). Black dots represent the included SNPs. Leave one-out analyses show the p-value and the beta estimate when perfroming the MR analyses without the SNP from the first column. I^2 is a measure of the strength of the included SNPs. P-values and the MR related parameters for each of the methods can be found in Table S8.

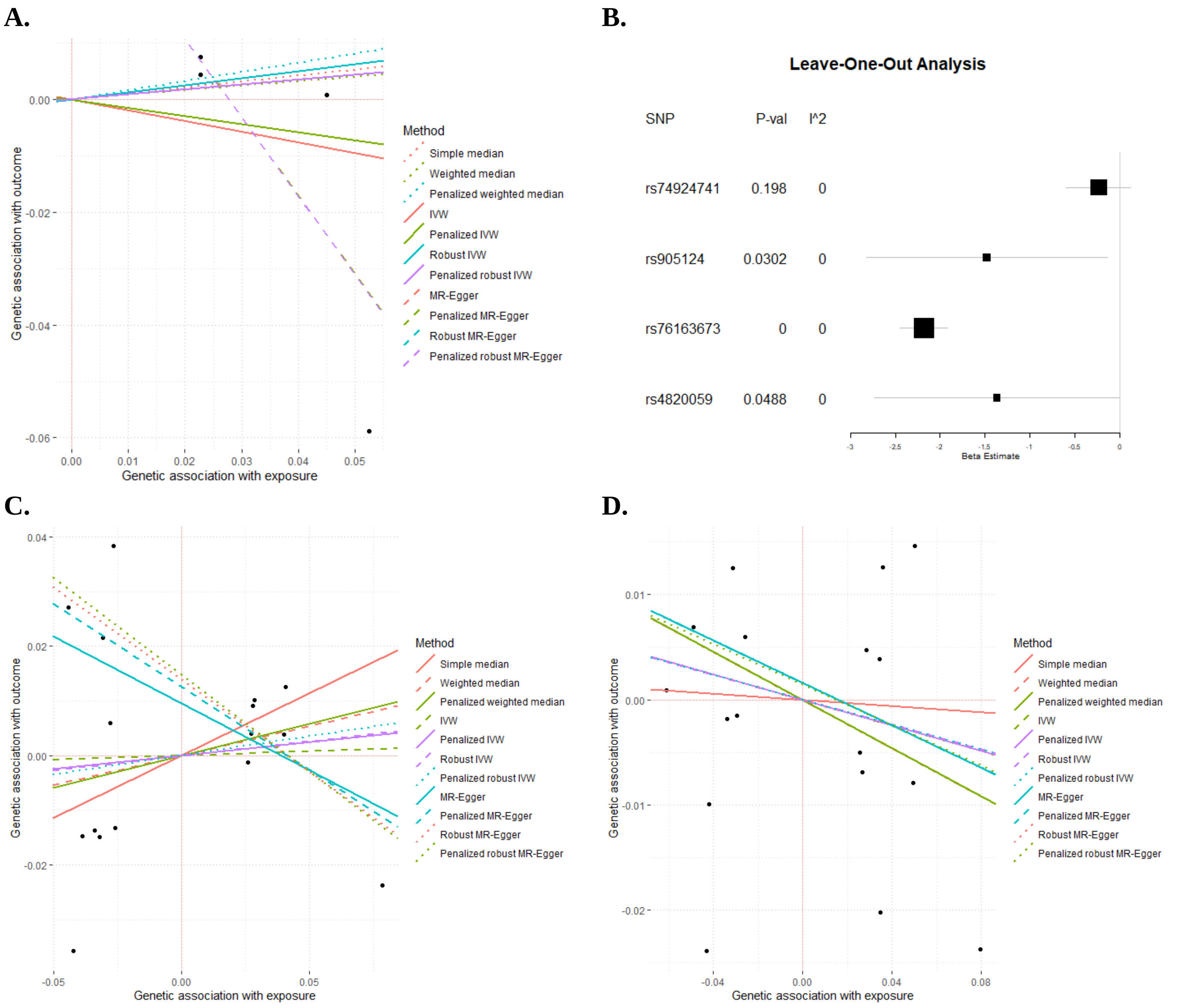

Supplement: Supplementary file 1 — Additional file 1. Supplemental Figures. [file 40478_2020_1072_MOESM1_ESM.pdf]
